# Cross‐compartment metabolic coupling enables flexible photoprotective mechanisms in the diatom *Phaeodactylum tricornutum*

**DOI:** 10.1111/nph.15685

**Published:** 2019-02-14

**Authors:** Jared T. Broddrick, Niu Du, Sarah R. Smith, Yoshinori Tsuji, Denis Jallet, Maxwell A. Ware, Graham Peers, Yusuke Matsuda, Chris L. Dupont, B. Greg Mitchell, Bernhard O. Palsson, Andrew E. Allen

**Affiliations:** ^1^ Division of Biological Sciences UC San Diego La Jolla CA 92093 USA; ^2^ Department of Bioengineering UC San Diego La Jolla CA 92093 USA; ^3^ Scripps Institution of Oceanography UC San Diego La Jolla CA 92093 USA; ^4^ J. Craig Venter Institute La Jolla CA 92037 USA; ^5^ Department of Environmental Bioscience Kwansei Gakuin University Sanda 669‐1337 Japan; ^6^ Department of Biology Colorado State University Fort Collins CO 80523 USA

**Keywords:** diatom, energy metabolism, genome‐scale modeling, photorespiration, flux balance, analysis, *Phaeodactylum tricornutum*

## Abstract

- Photoacclimation consists of short‐ and long‐term strategies used by photosynthetic organisms to adapt to dynamic light environments. Observable photophysiology changes resulting from these strategies have been used in coarse‐grained models to predict light‐dependent growth and photosynthetic rates. However, the contribution of the broader metabolic network, relevant to species‐specific strategies and fitness, is not accounted for in these simple models.
- We incorporated photophysiology experimental data with genome‐scale modeling to characterize organism‐level, light‐dependent metabolic changes in the model diatom *Phaeodactylum tricornutum*. Oxygen evolution and photon absorption rates were combined with condition‐specific biomass compositions to predict metabolic pathway usage for cells acclimated to four different light intensities.
- Photorespiration, an ornithine‐glutamine shunt, and branched‐chain amino acid metabolism were hypothesized as the primary intercompartment reductant shuttles for mediating excess light energy dissipation. Additionally, simulations suggested that carbon shunted through photorespiration is recycled back to the chloroplast as pyruvate, a mechanism distinct from known strategies in photosynthetic organisms.
- Our results suggest a flexible metabolic network in *P. tricornutum* that tunes intercompartment metabolism to optimize energy transport between the organelles, consuming excess energy as needed. Characterization of these intercompartment reductant shuttles broadens our understanding of energy partitioning strategies in this clade of ecologically important primary producers.

## Introduction

Understanding and predicting phytoplankton physiology in response to environmental changes has implications for ecological and industrial applications. Light is a dynamic environmental input that functions as both an energy source and a regulatory signal, affecting phenotype and cell physiology. Diatoms, important oceanic primary producers and attractive metabolic engineering candidates, have a diverse set of photoacclimation mechanisms (Falkowski & Owens, 1980; Wilhelm *et al*., 2014). Diatoms, including the model organism *Phaeodactylum tricornutum*, respond to light by adjusting the light‐harvesting pigment content per cell (Jakob *et al*., 2007), remodeling the light‐harvesting complexes, and reorganizing the thylakoid membrane (Lepetit *et al*., 2012). Diatoms can also dissipate excess light energy upstream of the photosynthetic reaction centers (e.g. nonphotochemical quenching (NPQ)), and the energy that flows through alternative electron pathways downstream of photosystem II (PSII) (Lepetit *et al*., 2012; Wagner *et al*., 2017). Overall, these strategies reduce damage to the photosynthetic apparatus (Lavaud *et al*., 2007), balance the ATP/NADPH ratio, and dissipate excess energy through futile metabolic reactions, such as the reduction of O_2_ to water (Nawrocki *et al*., 2015). Substantial energetic coupling between chloroplasts and mitochondria has recently been demonstrated in diatoms (Bailleul *et al*., 2015), raising the possibility that mitochondria function photoprotectively during conditions of excess light. However, the mechanisms facilitating this energy transfer are unknown.

The organization of primary metabolism in diatoms is distinct from that found in other phototrophic organisms, in part because of their unique evolutionary history (Bowler *et al*., 2008). For example, diatoms have enzymes for the energy‐producing lower half of glycolysis in the mitochondria whereas this pathway is restricted to the cytosol in other eukaryotes (Kroth *et al*., 2008; Río Bártulos *et al*., 2018). Photorespiration is another metabolic pathway that differs between diatoms and plants (Kroth *et al*., 2008) and may serve a photoprotective role. Under high‐light conditions, the centric diatom *Thalassiosira weissflogii* upregulates expression of photorespiration pathway genes, indicating that photorespiration occurs during photoacclimation (Schnitzler Parker *et al*., 2004). In plants, 2‐phosphoglycolate is recovered as glycerate through a series of metabolic reactions in the peroxisome and mitochondria, although this process is considered wasteful as it occurs at the expense of energy and fixed carbon (C; Wingler *et al*., 2000). In *P. tricornutum*, open questions remain regarding how photorespiration intersects with the broader metabolic network (Davis *et al*., 2017). Thus, the role of these metabolic pathways to facilitate photoprotection and cross‐compartment energy coupling has not been fully explored.

Genome‐scale metabolic models (GEMs) are bottom‐up depictions of cellular metabolism (O'Brien *et al*., 2013). GEMs are constructed using an established protocol (Thiele & Palsson, 2010) where mass‐ and charge‐balanced reactions are added until the model contains the metabolic capabilities encoded in the organism's genome. Growth is typically simulated by constraining uptake and excretion rates and defining the macromolecular composition of the cell, known as the biomass objective function (Feist & Palsson, 2010). The output is a set of quantitative reaction flux distributions that can generate the biomass components and facilitates hypothesis generation and testing, data analysis, and engineering. High‐quality GEMs of phototrophic microorganisms have emerged as powerful tools for characterizing whole‐cell metabolic fluxes and uncovering unique metabolic capabilities. Despite the unique phylogeny of diatoms, the GEM for *P. tricornutum* is one of the most comprehensive eukaryotic models (Levering *et al*., 2016). As GEMs are built on the underlying metabolic network and incorporate compartmental targeting of biochemical reactions, they are uniquely suited to characterize the chimeric nature of diatom metabolism. For example, the *P. tricornutum* GEM suggested ornithine‐mediated energy coupling between the chloroplast and mitochondria as an intercompartment reductant shuttle (Levering *et al*., 2016).

One persistent challenge with phototrophic GEMs relates to explicitly accounting for light absorption. Without constraints on light absorption, model solutions are inconsistent with cellular photophysiology. Phenotypes such as reductant partitioning (Halsey *et al*., 2013) and light‐dependent metabolic acclimation (Nymark *et al*., 2009) are inaccessible to such under‐constrained models. Measurement of photon absorption and biomass composition resulted in a quantitative assessment of light energy partitioning in *P. tricornutum* (Jakob *et al*., 2007). However, simple modeling frameworks that can approximate light‐dependent growth and photosynthetic rates (Platt *et al*., 1980; Kiefer & Mitchell, 1983; Moisan & Mitchell, 1999) omit intracellular metabolic reactions, including intercompartment reductant shuttles, necessary for bioprocess design of light‐driven bioproduct engineering. Because photoacclimation is essential for understanding light utilization and related physiology, it is critical to develop methodologies that explicitly model light‐dependent cellular metabolism (Broddrick *et al*., 2016). Thus, an open area of research is connecting representations of photophysiology with the metabolic network.

## Materials and Methods

Additional details can be found in the Supporting Information Methods S1–S5.

### Cell cultivation and growth monitoring

Axenic cultures of the marine diatom *P. tricornutum* (CCAP 1055/1) were cultivated in 1 l vertical tubular bioreactors submerged in a temperature‐controlled water tank at 20°C at 60, 120, 300 and 600 μmol photons m^−2^ s^−1^ on a 12 h : 12 h, light : dark cycle. Diatom cultures were mixed by CO_2_‐enhanced air, which was regulated by a Cole‐Parmer (Vernon Hills, IL, USA) gas flow controller to stabilize the culture pH at 8.1 ± 0.3. Inoculations were carried out in *f*/2 medium 3 d before the first sampling point, diluted to 2–6 × 10^5^ cells ml^−1^ and sampled over two light–dark cycles. Biomass accumulation was monitored with optical density at 750 nm (OD_750_) during light periods. Analytical measurements were conducted at the end of the light period, *c*. 9 h into the 12 h cycle. Additional details can be found in Methods S1.

### Pigment analysis by high‐performance liquid chromatography (HPLC)

Chlorophylls and carotenoids were measured by HPLC. Aliquots of 50 and 100 ml were filtered on 2.5 cm Whatman GF/F filters (Darmstadt, Germany) and extracted with 3 ml of 90% acetone for 24 h. Pigments were separated on a C18 reverse‐phase column (Econosphere 5 μm, 4.3 mm × 25 cm; Alltech, Columbia, MD, USA) and quantified using standards. Additional details can be found in Methods S1.

### Particulate organic C and particulate nitrogen (POC/PON)

After sampling, 10 ml of culture were filtered through a precombusted (500°C, 2 h) Whatman GF/F filter, wrapped in precombusted foil and dried at 60°C overnight. Samples were stored in a desiccator before being shipped to the Marine Science Institute at the University of California, Santa Barbara, for analysis in accordance with EPA 440.0 (Zimmermann *et al*., 1997).

### Plastid volume determination

*Phaeodactylum tricornutum* UTEX 642 (Austin, TX, USA) was cultured in 25 cm^2^ vented culture flasks (Nippon Genetics Co. Ltd, Tokyo, Japan) with *c*. 15 ml of *f*/2 artificial sea water (ASW) at 20°C. Cultures were acclimated to 10, 150 and 600 μmol photons m^−2^ s^−1^ of photosynthetically active radiation (PAR) from a white LED, determined with a Li‐Cor LI‐250A light probe. Cultures were sampled in early to mid‐exponential phase and imaged using a Leica TCS SP8 confocal microscope. Chl autofluorescence of representative cells was detected at 640–715 nm following laser excitation at 552 nm. The chloroplast volume was calculated from *z*‐stacked images of Chl autofluorescence using Fiji (Schindelin *et al*., 2012). The chloroplast volume was converted to lipid mass per cell using published values (Abida *et al*., 2015). Additional details can be found in Methods S2 and Table S8.

### Photophysiology constraints

Photophysiology constraints were based on an extension of photoautotrophic modeling of cyanobacteria (Broddrick *et al*., 2016). Briefly, the photon uptake flux was determined using the spectral distribution for the given light source at the experimental irradiance, the Chl*a*‐specific spectral absorption coefficient (a*_ph_(*λ*)), and the biomass fraction of Chl*a*. To fully capture the wavelength‐specific light–pigment interactions, we switched from PAR to quantum flux (QF) (Morel, 1978), which describes the total absorbed photon flux. The measured photosynthetic rates (oxygen evolution in this study; see Methods S1) were then fitted to equations for photosynthesis prediction (Platt *et al*., 1980; Aalderink & Jovin, 1997), using QF as the independent variable. Additional details can be found in Methods S3.

### Photoautotrophic simulations of cellular growth

Details on the simulations can be found in Methods S4. Briefly, the *P. tricornutum* GEM, iLB1025 (Levering *et al*., 2016), was updated with recent advances in diatom metabolic understanding (Table S1). For the sinusoidal culture, the model was simulated under a 12 h : 12 h, light : dark cycle, *f*/2 media, with 500 ml total culture volume and light from a white LED above the culture as previously reported (Jallet *et al*., 2016). For the photoacclimated cultures in the tubular bioreactors, the total culture volume was 800 ml and light was delivered from 360°. The 24 h simulation period was broken into 20 min intervals, with each interval considered to be at a pseudo‐steady state. For each interval, the total photon absorption of the culture was integrated, accounting for culture self‐shading; this QF value was set as the photon uptake (model reaction: EX_photon_e) and used to determine the oxygen evolution for the culture during the simulation interval (model reaction: EX_o2_e) as described in Broddrick *et al*. (2016). The biomass objective function was updated at the beginning of each interval in accordance with the derived dynamic biomass allocation (Table S2). The objective was maximization of cellular biomass. All simulations were performed using cobrapy (Ebrahim *et al*., 2013) in custom IPython notebooks (Pérez & Granger, 2007) with the default GLPK solver.

### Sensitivity analysis

Sensitivity to changes in the biomass composition (biomass component ratios) and photophysiology parameters (a*_ph_(*λ*), PAR, initial biomass DW, and maximum oxygen evolution) were determined by generating 1000 random perturbations for each parameter set (±0–50% and ±0–30% from the initial simulation value, respectively). A single 20 min biomass accumulation period was simulated and the resulting growth rate was compared with the default value. All simulations were performed using cobrapy (Ebrahim *et al*., 2013) in custom IPython notebooks (Fernando *et al*., 2007) with the default GLPK solver.

## Results and Discussion

In this study we leveraged a genome‐scale modeling approach to hypothesize photoprotective metabolic pathways in *P. tricornutum*. Starting from the published *P. tricornutum* GEM (Levering *et al*., 2016), we developed a modeling framework to recapitulate diurnal growth, incorporating time‐course biomass measurements (Jallet *et al*., 2016). We then applied this framework to model photoautotrophic growth in *P. tricornutum* acclimated to four light intensities spanning an order of magnitude. The simulation outputs were validated against experimental values. Finally, photoprotective pathways were hypothesized based on the reaction flux predictions at each light intensity. A summary of the framework generation and simulations along with the relevant steps, inputs, outputs, validation metrics, and references can be found in Table 1.

**Table 1:** Summary of the framework generation and simulations

Model framework	Input^a^	Genome‐scale model of *Phaeodactylum tricornutum*	Levering *et al*. (2016)
		Biomass allocation over a diurnal cycle	Jallet *et al*. (2016)
		Photophysiology (O_2_ evolution, a*, pigments, plastid size)	Jallet *et al*. (2016); this study
	Output	Circadian growth model of *P. tricornutum*	
	Validation^b^	TOC, TN, biomass accumulation rates, transcriptomics	Jallet *et al*. (2016); Smith *et al*. (2016)
Photoacclimation simulations	Input^a^	Circadian growth model of *P. tricornutum*	This study (model framework)
		Photophysiology (O_2_ evolution, a*, pigments, plastid size)	This study (experimentally determined)
		Biomass allocation under a square‐wave light regime	This study (experimentally determined)
	Output	Growth rate predictions, biomass accumulation rates, reaction fluxes	
	Validation^b^	Growth rate, POC, PON, Chl*a* concentration, transcriptomics	This study; Smith *et al*. (2016); Levering *et al*. (2017)

References for inputs or validation data not generated in this study are indicated. a*, Chl*a*‐normalized optical absorption cross‐section; TOC, total organic carbon; TN, total nitrogen; POC, particulate organic carbon; PON, particulate organic nitrogen.

a Inputs include the underlying model and parameters required for simulation.

b Validation metrics compare simulation results with experimental data.

### Generating the simulation framework

We first generated a framework that models photoautotrophic growth in light–dark cycles. The requisite inputs and outputs are captured by three groups of parameters: photophysiology, biomass composition, and dynamic allocation of fixed C to different biomass components over the light period. A recent investigation into *P. tricornutum* grown in a sinusoidal light regime determined most of these requirements experimentally (Jallet *et al*., 2016).

The photophysiology parameters consist of the oxygen‐based net photosynthetic rate (*P*), the Chl*a‐*specific absorption coefficient (a*_ph_(*λ*)), and the Chl*a* concentration (Broddrick *et al*., 2016). Photosynthesis vs irradiance curves and the Chl*a* were reported in Jallet *et al*. (2016), but a*_ph_(*λ*) was not reported. As a*_ph_(*λ*) is related to pigment content, we approximated a*_ph_(*λ*) by taking the absorption coefficient of a block‐light *P. tricornutum* culture with a Chl*a* content per cell similar to that reported for the sinusoidal culture (0.28 vs 0.27 pg per cell, respectively, at *T* = 9 h; Fig. S1a,c). As the approximated a*_ph_(*λ*) value underestimated the light attenuation through the culture path length, it was adjusted to fit the experimental values (Fig. S1b).

**Figure 1:**
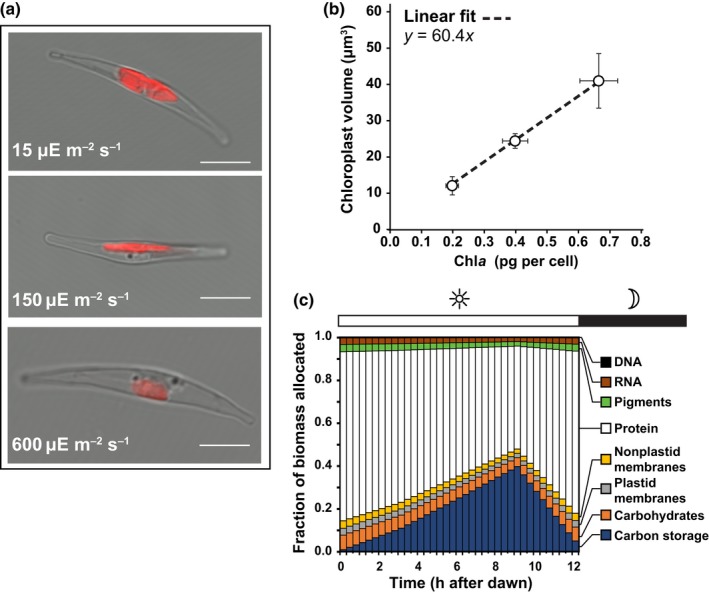
Cellular composition changes as a function of irradiance as well as progression through the light period. (a) Confocal microscopy images of representative *Phaeodactylum tricornutum* cells acclimated to 15, 150 and 600 μmol photons m^−2^ s^−1^ of photosynthetically active radiation (PAR) (in the figure, μE = μmol photons). Chl autofluorescence is indicated in red. The scale bar in white is 5 μm. (b) Correlation between calculated chloroplast volume and experimental Chl*a* concentration. Vertical error bars are the standard deviation of at least five individual cell measurements per PAR intensity. Horizontal error bars are the standard deviation of three biological replicates at each PAR intensity. (c) Biomass allocation of the major cellular components across the light period, determined in this study, for *P. tricornutum* cultured under a sinusoidal light regime at full solar irradiance derived from values reported in Jallet *et al*. (2016) and this study. Values were used to parameterize the simulation of sinusoidal circadian growth.

The biomass composition defines which macromolecules (e.g. protein, pigments) are synthesized and in what proportion for a cell to grow. As these ratios vary across light environments, we approximated the resources shifted to light harvesting as a function of photoacclimation (Fig. 1a). Using a derived equation (Eqn S1 in Methods S2) to approximate plastid volume, validated against *z*‐stacked images of Chl autofluorescence (Fig. S2; Table S3), we determined the mean volumes of the plastid to be 40.5 ± 7.5, 23.9 ± 2.0 and 11.6 ± 2.5 μm^3^ at 0.67 ± 0.06, 0.40 ± 0.04 and 0.20 ± 0.02 pg Chl*a* per cell, respectively (Fig. 1b). As these variables were positively correlated (Fig. 1b), we approximated the plastid volume to be 11.5 μm^3^ at dawn (*T* = 0) for the sinusoidal light culture in Jallet *et al*. (2016). Using published values for *P. tricornutum* (Abida *et al*., 2015), the calculated volume corresponded to a photosynthetic lipid content of 4.5% of the total biomass at dawn (Table S2). Although the plastid volume was determined using a different strain of *P. tricornutum* (UTEX642), the pigment concentration as a function of PAR was consistent with studies in CCAP 1055/1 (Nymark *et al*., 2009).

**Figure 2:**
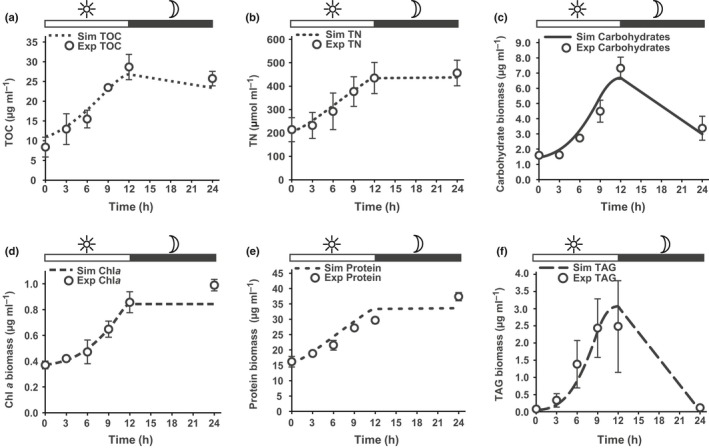
Model simulations vs experimental values for *Phaeodactylum tricornutum* cultured under a diurnal sinusoidal light regime. (a) Total organic carbon (TOC) content in the culture biomass. (b) Total nitrogen (TN) content in the culture biomass. (c) Total carbohydrate content in the culture biomass, including structural and storage carbohydrates. (d) Total Chl*a* content in the culture biomass. (e) Total protein content in the culture biomass. (f) Total triacylglycerol (TAG) content in the culture biomass. Light and dark periods are indicated above the plot. Error bars on the experimental values are the standard deviations reported with the published data (Jallet *et al*., 2016).

The final parameter captured resource partitioning across the light period. We generalized the biomass partitioning observed for the sinusoidal light regime by assuming all biomass components increase linearly through the light period except for Chl*a* and C storage compounds (triacylglycerol (TAG) and chrysolaminarin). These components are characterized by an initial lag followed by a rapid increase in resource allocation (Fig. 2c,d,f). Using this dynamic, we derived biomass partitioning coefficients that quantify the fraction of resources (e.g. C, N) allocated to each biomass component over the light period (Fig. 1b; Table S2). Although these coefficients were based on experimental results and are not *de novo* model predictions, they do characterize an underlying regulatory mechanism inherent to circadian metabolism. With this final parameter defined, the simulation framework was complete.

We validated the framework by simulating circadian growth of *P. tricornutum* under a sinusoidal light regime over a 12 h : 12 h, light : dark cycle and compared the results with published experimental values. Combining the experimental data with the model biomass values reconstructed the C balance for most of the time points reported (Jallet *et al*., 2016) (Table S4). There was a lack of C balance in the late light period, visible as the difference between TOC in our reconstructed biomass and the experimentally determined value. This suggested the model's biomass composition underestimated the DW of components lacking experimental data (e.g. RNA). The simulation results accurately recapitulated the light period accumulation of all major biomass components (Fig. 2a–f). For the dark period, accumulation of Chl*a* and protein was underestimated (Fig. 2d,e).

We performed a sensitivity analysis, which is a deliberate perturbation of the model inputs away from their set values, for the photophysiology constraints and biomass composition. This analysis highlighted how inaccuracies in experimental data or derived parameters affect the predicted growth rate. The oxygen evolution rate had the most dramatic impact (*F* = 1.0, *P* = 10^−99^; Fig. S3d). PAR and initial biomass both had statistically significant, albeit minor, impacts (Fig. S3a,b), whereas a*_ph_(*λ*) had an insignificant effect (Fig. S3c). Sensitivity analysis for the biomass composition indicated that protein and pigment biomass had a significant impact on growth rate (Fig. S4e,f). Pigment content is linked to the oxygen evolution rate, the dominant biophysical constraint. As protein constituted the most cellular biomass (*c*. 78%; Table S2), perturbations in this component dramatically affect the remaining biomass components, including pigments. Therefore, biologically realistic simulation outputs are dependent on accurately constraining oxygen evolution.

The simulation framework was sufficient to recapitulate the biomass accumulation in the sinusoidal‐light culture, but it did not accurately predict the dark period Chl*a* accumulation. The data reported by Jallet *et al*. (2016) showed dark‐period accumulation of Chl*a* despite the absence of a light‐independent protochlorophyllide oxidoreductase in the annotated genome of *P. tricornutum* (Hunsperger *et al*., 2016). This observation suggests that a pool of protochlorophyllide is biosynthesized during the light period and converted into Chls during the dark period, possibly restoring pigment levels after dilution as a result of cell division. Additionally, the model underestimated protein biomass accumulation in the dark period (Fig. 2e). The simulations indicated that intracellular C storage compounds were insufficient to satisfy maintenance energy requirements while also generating additional protein biomass. This result suggests that dark‐period metabolism in *P. tricornutum* is fueled by biomass components in addition to TAGs and chrysolaminarin. These results demonstrate gaps in our understanding of diatom dark metabolism and highlight the value of structuring assumptions into a modeling framework where they can be evaluated for validity.

### Cultivation and photophysiology

Next, we generated the modeling inputs necessary to investigate photoprotective metabolic pathways in *P. tricornutum*, which was cultured at PAR values of 60, 120, 300 and 600 μmol photons m^−2^ s^−1^, covering the range of sub‐ to postsaturating illumination intensities. Cell‐specific growth rates, measured by optical density at 750 nm (OD_750_), increased with increasing PAR from 0.05 to 0.11 h^−1^ (Fig. S5a). Cultures were sampled late in the light period (*T* = 9 h) to facilitate comparison of C accumulation (POC), N assimilation (particulate organic N, PON) and Chl*a* accumulation throughout the light period with model simulations. The C : Chl*a* ratio increased continuously, doubling between 60 and 600 μmol photons m^−2^ s^−1^, indicating an increase in biomass resources directed to light harvesting at low PAR values (Table S5). The light‐harvesting pigments Chl*a*, Chl*c*, and fucoxanthin decreased with increasing PAR, whereas the ratios between these pigments were consistent. However, the photoprotective pigments beta‐carotene and the NPQ‐related xanthophyll pigments diatoxanthin and diadinoxanthin increased with rising PAR, relative to Chl*a*, suggesting an increase in energy dissipation. These results (Table S5) are consistent with previous observations (Jakob *et al*., 2007; Nymark *et al*., 2009).

Using the derived relationship between chloroplast volume and Chl*a* (Fig. 1a), we approximated the chloroplast volumes to be 40.1, 35.2, 19.6 and 17.6 μm^3^ at 60, 120, 300 and 600 μmol photons m^−2^ s^−1^, respectively. The calculated volumes correspond to a photosynthetic lipid content ranging from 3.0% to 8.8% of total biomass at 600 and 60 μmol photons m^−2^ s^−1^, respectively (Table S6). These results facilitated a proper accounting of macromolecule biosynthesis that was incorporated into our model simulations (see later).

**Figure 3:**
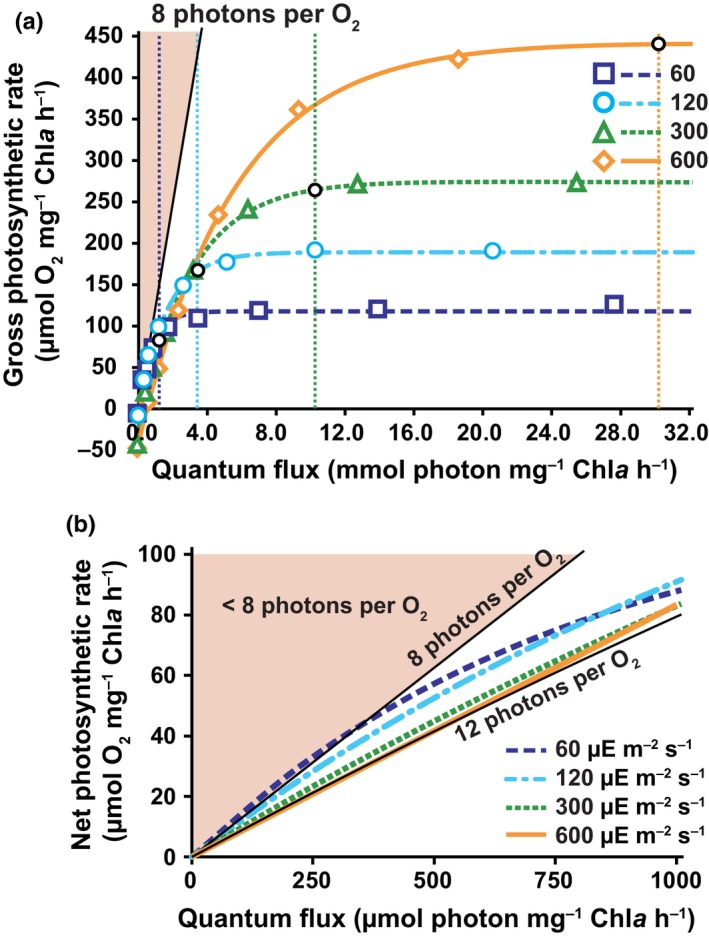
Photoacclimation results in efficient photosynthesis across experimental photosynthetically active radiation (PAR) values. (a) Gross photosynthesis vs quantum flux (*P* vs QF) across the experimental PAR values. Experimental PAR values in units of μmol photon m^−2^ s^−1^ are indicated in the legend. Vertical dashed lines indicate the respective QF at each of the experimental PAR values. The black, open circles indicate the QF and photosynthetic rate used to calculate the experimental quantum requirement. The shaded region indicates the theoretical minimum quantum requirement lower bound of eight photons per oxygen evolved. (b) Expanded view of the initial slope of net *P* vs QF. Experimental PAR values are indicated in the legend. The shaded region indicates the theoretical minimum quantum requirement lower bound of eight photons per oxygen evolved. A line is displayed representing a minimum quantum requirement of 12 photons per oxygen evolved. (In the figure, μE = μmol photons.)

The Chl*a*‐specific absorption coefficient and Chl*a*‐specific gross photosynthetic oxygen evolution (P) vs PAR were measured for each condition and converted to P vs QF (Figs 3a, S6, S7). This metric describes the actual photon absorption rate, as compared with PAR, which is the photon delivery rate. We approximated the minimum quantum requirement (MQR) (Dubinsky *et al*., 1986) at the acclimated irradiances using the initial slope of the net P vs QF curves. (Fig. 3b). The MQR increased with PAR and the photon requirement at 60 μmol m^−2^ s^−1^ slightly below the theoretical limit of eight photons per O_2_ molecule. The MQR never exceeded 12 photons per O_2_.

Using fast repetition‐rate fluorometry (FrrF), we determined the maximum PSII light‐capture efficiency (*F*_v_/*F*_m_) to be similar across all conditions (*c*. 0.55; Fig. S5b). This *F*_v_/*F*_m_ value corresponded to an MQR of 11.3 photons per O_2_ molecule, assuming *z*‐scheme oxygen evolution requiring four photons performing photochemistry at both PSII and PSI. It has been shown that *F*_v_/*F*_m_ determined by FrrF is *c*. 20% lower than values from pulse‐amplitude‐modulation (PAM) fluorometry (Suggett *et al*., 2003). Therefore, PAM‐derived MQR would equate to *c*. 10 photons per O_2_. Both values were higher than those calculated from the initial slope of the *P* vs QF curve. Previous studies have yielded MQRs of < eight photons per molecule for O_2_ evolution in *P. tricornutum* (Osborne & Geider, 1987) and low electron requirements for C fixation in other diatoms (Morelle & Claquin, 2018). Our modeled data are consistent with previous findings, but they do point to an inconsistency between theoretical photosynthesis rates and their experimental/modeled determination in diatoms.

In contrast to the MQR determined for the initial slope of the *P* vs QF curve, the quantum requirement at the experimental quantum flux values suggested substantial intracellular consumption of photochemically produced O_2_. These values were 11.4, 21.0, 39.3 and 67.9 photons per O_2_ evolved at 60, 120, 300 and 600 μmol photons m^−2^ s^−1^, respectively (Fig. 3a). To investigate the validity of this prediction, light‐dependent oxygen consumption and production measurements were performed using membrane inlet mass spectrometry (MIMS). Cells acclimated to, and subsequently illuminated with, 60 and 600 μmol photons m^−2^ s^−1^ had oxygen consumption rates in the light that were 35 ± 5% and 20 ± 2% (average ± SD, *n* = 3) of gross oxygen evolution rates, respectively (Table S7; Methods S5). These measured intracellular O_2_ consumption rates are consistent with our quantum requirement predictions.

### Simulation of *P. tricornutum* photoacclimated to various PAR intensities

Genome‐scale flux balance analysis can hypothesize cross‐compartment reductant shuttles responsible for the observed intracellular O_2_ consumption, but the model must first recapitulate the biophysical characteristics of the phenotype. We validated our simulation framework's ability to recapitulate photoautotrophic growth in *P. tricornutum* acclimated to the four light intensities.

**Figure 4:**
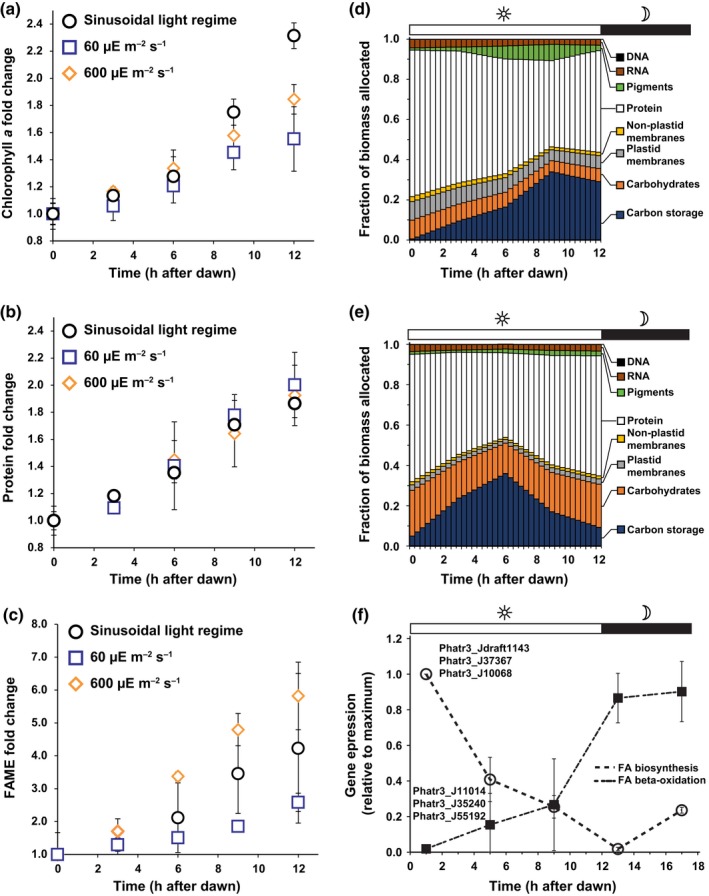
Biomass allocation dynamics for *Phaeodactylum tricornutum* acclimated to different light regimes. Fold‐change in Chl*a* (a), protein (b) and fatty acid methyl esters (FAMEs) (c) over the light period for cells acclimated to a sinusoidal light regime at full solar irradiance (circles), and square‐light regimes at low (squares, 60 μmol photons m^−2^ s^−1^) and high (diamonds, 600 μmol photons m^−2^ s^−1^) irradiance. Error bars represent the standard deviation of three replicates for the square‐wave light regime and the standard deviations reported with the sinusoidal data (Jallet *et al*., 2016). (d) Biomass allocation of the major cellular components across the light period, determined in this study, for *P. tricornutum* cultured under a square‐wave light regime at a photosynthetically active radiation (PAR) value of 60 μmol photons m^−2^ s^−1^. Values were used to parameterize the genome‐scale model simulations. (e) Biomass allocation of the major cellular components across the light period, determined in this study, for *P. tricornutum* cultured under a square‐wave light regime at a PAR value of 600 μmol photons m^−2^ s^−1^. Values were used to parameterize the genome‐scale model simulations. (f) Mean transcript abundance, relative to maximum, for genes associated with fatty acid biosynthesis (open circles) and fatty acid degradation (closed squares) from *P. tricornutum* as reported in Smith *et al*. (2016). Maximum was *T* = 0 for biosynthesis genes and *T* = 17 for beta‐oxidation genes. Error bars are the standard deviation of at least three biological replicates as reported (Smith *et al*., 2016) of three representative genes for each pathway. FA biosynthesis‐Phatr3_Jdraft1143: 3‐hydroxyacyl‐[acyl‐carrier‐protein] dehydratase, Phatr3_J37367: 3‐oxoacyl‐[acyl‐carrier‐protein] synthase, Phatr3_J10068: enoyl‐[acyl‐carrier‐protein] reductase; FA degradation‐Phatr3_J11014: Acyl‐CoA dehydrogenase, Phatr3_J35240: 3‐hydroxacyl‐CoA dehydrogenase, Phatr3_J55192: Enoyl‐CoA hydratase. (In the figure, μE = μmol photons.)

We parameterized the simulation framework with the experimentally determined photophysical constraints (*P* vs QF, a*_ph_(*λ*), Chl*a*; Figs 3a, S6) at each PAR intensity. Based on Chl*a* content per cell and the calculated photosynthetic lipid content (see above in ‘Generating the simulation framework’), biomass allocated to photosynthetic components ranged from 6.6% to 14.0% of total biomass at 600 and 60 μmol photons m^−2^ s^−1^, respectively (Table S6). For the condition‐specific biomass component ratios, we investigated the biomass allocation dynamics in a square‐wave light regime. Sensitivity analysis of the model framework based on the sinusoidal light regime indicated Chl*a* and protein content significantly affected the accuracy of predictions (Fig. S4). To investigate differences in the allocation dynamics between sinusoidal and square‐wave light regimes, we experimentally determined the Chl*a* and protein accumulation across the light period for the low‐light (LL, 60 μmol photons m^−2^ s^−1^) and high‐light (HL, 600 μmol photons m^−2^ s^−1^) conditions. Additionally, we characterized lipid content as fatty acid methyl esters (FAMEs) to compare the C‐storage dynamics. There were significant differences in the allocation dynamics of Chl*a* and FAME accumulation but not for protein biomass (Fig. 4a,b,c). Similar to our analysis for the sinusoidal data, we used these dynamics to derive square‐wave light regime partitioning coefficients for all biomass components (Fig. 4d,e; Table S2). These results highlight significant differences in biomass allocation dynamics between light regimes and irradiances. Predicted storage C allocation was consistent with the patterns of gene expression for fatty acid beta‐oxidation and biosynthesis as reported in a previous investigation into diel gene expression in *P. tricornutum* (Smith *et al*., 2016) (Fig. 4f).

Photoautotrophic growth at each photoacclimated irradiance across a 12 h light period was simulated according to the validated framework (Table S2). As the cultures were supplied with CO_2_‐enhanced air, the dissolved inorganic carbon source was set to CO_2_, simulating a suppressed carbon concentrating mechanism (CCM) (Nakajima *et al*., 2013). The model simulates mass accumulation, and therefore OD_750_ values were converted to mg DW using condition‐specific conversion factors (Methods S4). As outlined earlier, we derived biomass partitioning coefficients based on the LL and HL data (Fig. S5c,d). For the intermediate light intensities, we interpolated between the LL and HL dynamics and tested if this assumption could recapitulate the observed growth rate and biomass content.

**Figure 5:**
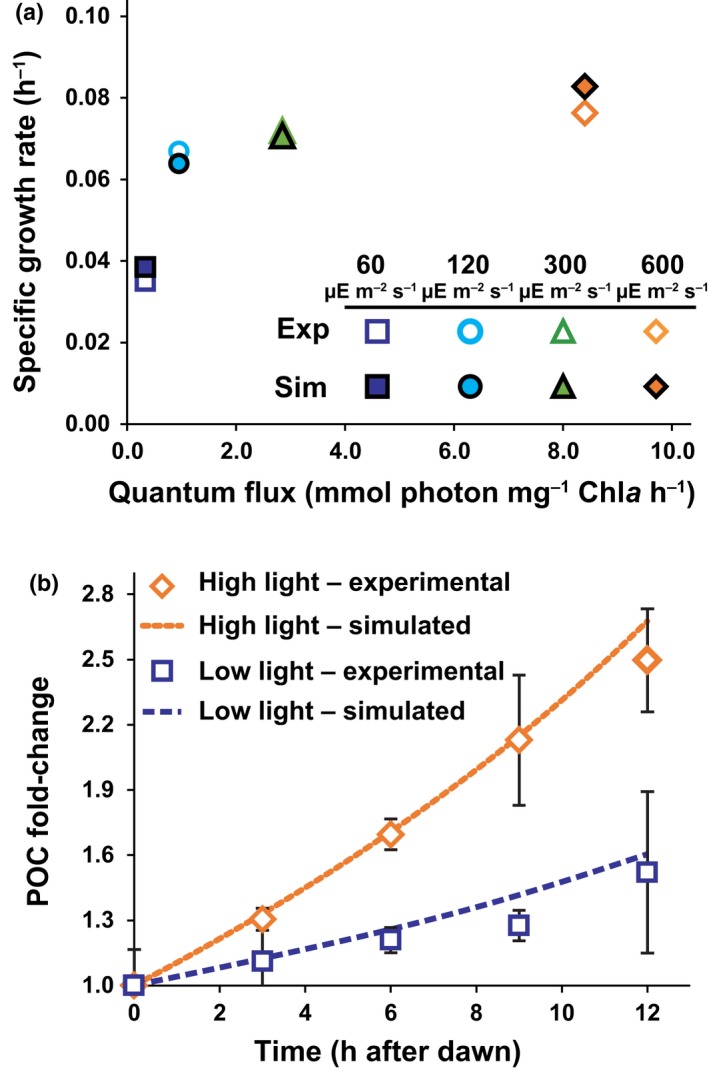
Model simulations vs experimental values for *Phaeodactylum tricornutum* cultured under various photoacclimated photosynthetically active radiation (PAR) intensities. (a) Growth rate comparison, based on biomass accumulation, between measured (open markers) and simulated values (closed markers). PAR values are indicated in the legend. (In the figure, μE = μmol photons.) (b) Rate of biomass increase, relative to dawn, for the high‐light (600 μmol photons m^−2^ s^−1^) and low‐light (60 μmol photons m^−2^ s^−1^) cultures. Markers indicate experimental values, and the lines are simulation values. Error bars are the standard deviation of at least three biological replicates.

**Figure 6:**
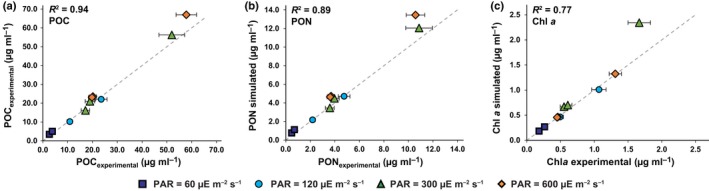
Model simulations vs experimental values for *Phaeodactylum tricornutum* biomass components cultured under various photoacclimated photosynthetically active radiation (PAR) intensities. (a) Particulate organic carbon (POC) content comparison between measured and simulated values in mass per culture volume. Error bars are the combined standard deviations of two technical replicates and cell count measurements. (b) Particulate organic nitrogen (PON) content comparison between measured and simulated values in mass per culture volume. Error bars are the combined standard deviations of two technical replicates and cell count measurements. (c) Chl*a* content comparison between measured and simulated values in mass per culture volume. Error bars represent the standard deviation of cell count measurements. Photoacclimated PAR values are indicated by color and marker shape. The dashed line indicates the line of perfect agreement between the experimental and simulated values. The coefficient of determination (deviation from perfect agreement, *R* ^2^) is indicated for each biomass component. (In the figure, μE = μmol photons.)

The simulated growth rates, based on biomass accumulation, were within 10% of the experimental values at all PAR intensities (Fig. 5a). Additionally, simulated C accumulation matched the experimental dynamics (Fig. 5b). Upon converting the values for POC, PON and Chl*a* from content per cell to mg ml^–1^ (*n* = 2 for 60 and 120 μmol photons m^−2^ s^−1^, *n* = 3 for 300 and 600 μmol photons m^−2^ s^−1^), comparison with the model predictions showed good agreement for these biomass components (Fig. 6a–c). Overall, the model accurately recapitulated the quantitative differences in growth rate and biomass accumulation. These results do not represent a *de novo* prediction by the model, as they required experimental parametrization. Notably, however, they do suggest that the Chl*a* and storage compound accumulation dynamics are sufficient to characterize the photoacclimation phenotype as well as highlight the ability of genome‐scale modeling to contextualize and extend experimental data.

### Metabolic pathway usage between low‐ and high‐light acclimation

**Figure 7:**
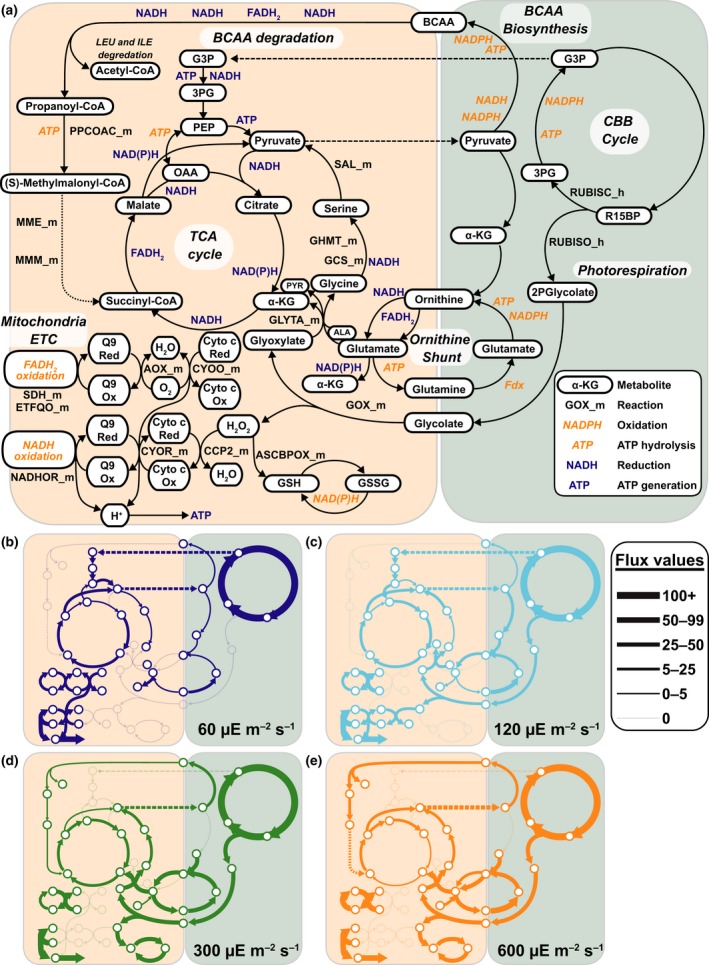
Metabolic reconfiguration at various photoacclimated photosynthetically active radiation (PAR) values. (a) Overview of metabolic coupling between the mitochondria and chloroplast in *Phaeodactylum tricornutum*. Dashed lines between the compartments indicate transport of metabolites. The dotted line represents a disputed metabolic pathway (see main text). (b–e) Predicted metabolic pathway usage and reaction flux for cultures acclimated to various PAR intensities. Line thickness corresponds to metabolic reaction flux normalized to 100 units of Rubisco carboxylase flux. Color indicates the PAR value according to the figure legend. 2Pglycolate, 2‐phosophoglycolate; 3PG, 3‐phosphoglycerate; α‐KG, alpha‐ketoglutarate; ALA, alanine; BCAA, branched‐chain amino acid; CBB, Calvin–Bensen–Bassham; Cyto, cytochrome; ETC, electron transport chain; G3P, glyceraldehyde‐3‐phosphate; GSH, glutathione (oxidized); GSSG, glutathione (reduced); Ox, oxidation; Q9, Ubiquinone; PYR, pyruvate; R15BP, ribulose‐1,5‐bisphosphate; Red, reduction; TCA, tricarboxylic acid. Reaction abbreviations are in BiGG format (bigg.ucsd.edu) and correspond to the abbreviations used in the model (Supporting Information Dataset S1). A more complete metabolic map and flux data for all model reactions can be found in the Dataset S1. (In the figure, μE = μmol photons.)

Pathway‐specific metabolic usage under various photoacclimation conditions was explored using the validated simulation framework developed above. Without additional constraints, there are multiple, mathematically equivalent solutions for a metabolic network as large and interconnected as that of *P. tricornutum*. We explored the most parsimonious solution (Lewis *et al*., 2010) for hypotheses related to reductant shuttles in *P. tricornutum*. To compare differential pathway usage independent of growth rate, the metabolic fluxes were normalized to 100 units of Rubisco carboxylase flux (Fig. 7a–e; Dataset S1).

At the lowest light condition simulated (60 μmol photons m^−2^ s^−1^), chloroplast glyceraldehyde‐3‐phosphate (G3P) shuttled reductant and ATP to the mitochondria. After being oxidized by mitochondrial glycolysis, the C returns to the chloroplast as pyruvate (Fig. 7a,b). To a lesser degree, C skeletons and reductant from the chloroplast are also delivered to mitochondria as ornithine synthesized in the plastid and returned as glutamine through a previously hypothesized shunt mechanism (Levering *et al*., 2016) (Fig. 7b). At the lowest light condition, the model suggested over half of the reductant funneled to the mitochondrial electron transport chain (ETC) was used to generate ATP (Fig. 7b).

**Figure 8:**
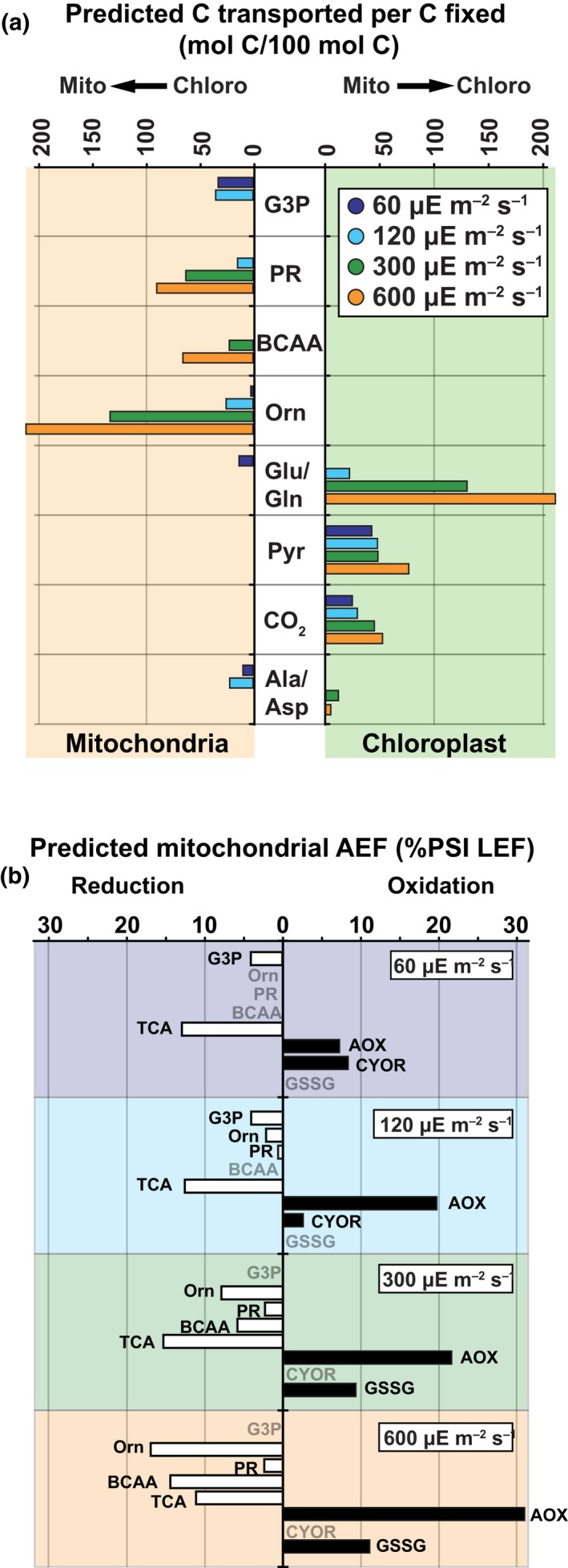
Alternate electron flows (AEF) in *Phaeodactylum tricornutum* as a function of photoacclimated photosynthetically active radiation (PAR) values. (a) Carbon cycling between the chloroplast and mitochondria. Metabolites shuttled to the mitochondria are indicated in the left‐hand shaded region. Carbon recycling metabolites returned the chloroplast are indicated in the right‐hand shaded region. Units are given in mol carbon (100 mol CO_2_)^–1^ fixed by Rubisco. Photoacclimated PAR values (μmol photons m^−2^ s^−1^) are indicated by bar color. (b) Mitochondrial consumption of photosynthetically generated electrons. Metabolites that reduce mitochondrial electron carriers are indicated on the left‐hand side of the vertical axis. Reactions that consume mitochondrial reductant are indicated on the right. Values are given in percent photo‐system I linear electron flow (%PSI LEF). PAR values (μmol photons m^−2^ s^−1^) are indicated by the shaded regions. AOX, alternative oxidase; BCAA, branched‐chain amino acid; CYOR, cytochrome c reductase; G3P, glyceraldehyde‐3‐phosphate; GSSG, glutathione reduction; Orn, ornithine; PR, photorespiration; Pyr, pyruvate; TCA, tricarboxylic acid cycle. Amino acids are shown using their three‐letter code. (In the figure, μE = μmol photons.)

Metabolic flux simulations at the highest light conditions (300 and 600 μmol photons m^−2^ s^−1^) were similar (Fig. 7d,e) but distinct from flux predictions in low light (Fig. 7b), with an intermediate configuration observed at 120 μmol photons m^−2^ s^−1^ (Fig. 7c). At high light, flux through the metabolic network shifted to maximize excess energy consumption. Mitochondrial ETC was configured for energy dissipation with increased flux through alternative oxidase (AOX) with no flux through ubiquinol‐cytochrome *c* oxidoreductase (Complex III) or cytochrome *c* oxidase (complex IV) (Figs 7c,d, 8b). There was increased flux though the ornithine shunt at higher light and the shunt was almost 100% cyclic, suggesting a configuration to maximize energy export out of the chloroplast (Fig. 8a). Branched‐chain amino acid (BCAA) synthesis in the chloroplast and catabolism in the mitochondria were both elevated, delivering reductant between the organelles for processing in the mitochondrial ETC. C skeletons from BCAA degradation were converted to pyruvate through the back half of the TCA cycle for transport to the chloroplast, sustaining this shuttle. Another major difference at high light was the increase in photorespiration, resulting in elevated flux of glycolate from the chloroplast to mitochondria (Fig. 7c,d). Glycolate is oxidized in the mitochondria and metabolized to pyruvate for export to the chloroplast, with the final step catalyzed by serine ammonia lyase (SAL_m; Fig. 7a). Ascorbate peroxidase metabolizes peroxides produced during glycolate oxidation. Elevated photorespiration at high light is accompanied by reduced flux of G3P from the chloroplast and reduced flux through the mitochondrial glycolysis pathway. At all PAR intensities, intercompartment transport of the amino acids alanine and aspartate balanced C and N requirements (Fig. 8a).

The model predicted that the mitochondrial alternative oxidase (AOX_m) consumed 15–30% of the photosynthetically derived electrons that enter the broader metabolic network (PSI flux minus cyclic electron flow; Fig. 8b); which is consistent with the MIMS results (Table S7). However, this comparison also suggests that the model underestimated nonphotochemical dissipation of captured light energy at high light. Additionally, the photoprotective function suggested by the simulations are consistent with decreased photosynthetic rates observed in AOX_m knockout lines (Murik *et al*., 2019).

### Photorespiratory C may be recovered as pyruvate in *P. tricornutum*

**Figure 9:**
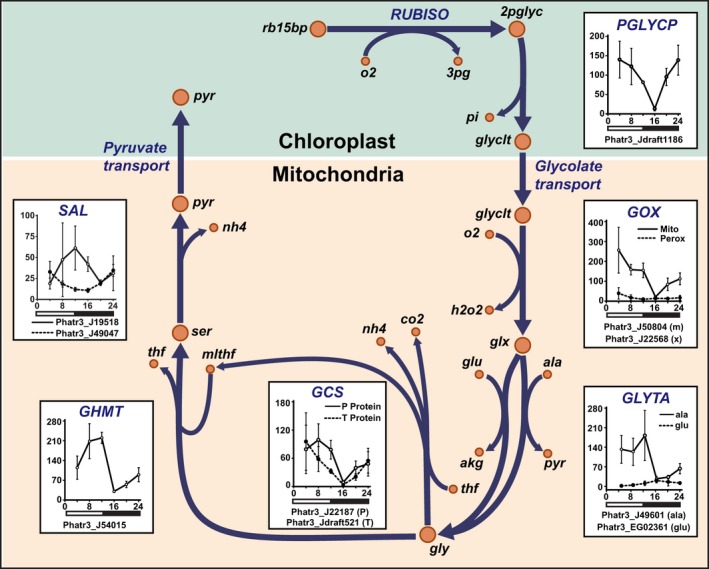
Mitochondrial glycolate oxidase participates in photorespiration. Model‐predicted photorespiration reaction flux between the chloroplast and mitochondria in *Phaeodactylum tricornutum*, and the correlated gene expression levels (reads per kilobase of transcript per million mapped reads) during a diel cycle (Smith *et al*., 2016). Error bars represent the standard deviation of at least three biological replicates as reported in Smith *et al*. (2016). Reaction and metabolite abbreviations are in BiGG format (http://bigg.ucsd.edu/) and correspond to the abbreviations used in the model (Dataset S1). 2pglyc, 2‐phosphoglycolate; 3pg, 3‐phosphoglycerate; akg, 2‐oxoglutarate; ala, alanine; co2, carbon dioxide; GCS, glycine cleavage system; GHMT, glycine/serine hydroxymethyl transferase; glu, glutamate; glyclt, glycolate; glx, glyoxylate; GLYTA, glycine/alanine transaminase; gly, glycine; GOX, glycolate oxidase; h2o2, hydrogen peroxide; m, mitochondria localized; mlthf, methylene tetrahydrofolate; nh4, ammonium; o2, oxygen; PGLYCP, 2‐phosphoglycolate phosphatase; pi, inorganic phosphate; pyr, pyruvate; rb15bp, ribulose‐1,5‐bisphosphate; RUBISO, ribulose‐1,5‐bisphosphate oxidase; SAL, serine ammonia lyase; ser, serine; thf, tetrahydrofolate; x, peroxisome localized.

Simulations indicated that glycolate is processed via the mitochondrial glycolate oxidase (GOX_m). In plants, glycolate oxidation occurs in the peroxisome (Bauwe *et al*., 2012); however, there is recent evidence that the mitochondrial GOX participates in the photorespiratory cycle in *P. tricornutum* despite the presence of a peroxisomal glycolate oxidase (GOX_x) (Schmitz *et al*., 2017). Transcript‐level expression of both GOX enzymes in *P. tricornutum* using published data (Smith *et al*., 2016) provided further evidence for this mitochondrial pathway. The transcript abundance of GOX_m was 40 times higher than GOX_x on average, and GOX_m was coexpressed with known photorespiration genes, including glycine decarboxylase and phosphoglycolate phosphatase (Figs 9, S8). Taken together, the flux predictions are consistent with biochemical evidence (Schmitz *et al*., 2017), gene expression in *P. tricornutum* (Smith *et al*., 2016) and the centric diatom *T. pseudonana* (Davis *et al*., 2017).

In our simulations, serine‐ammonia lyase (SAL) closes the photorespiration loop to return C skeletons to the chloroplast as pyruvate. This is different from plants where C skeletons are returned as 3‐phosphoglycerate (3PG). In addition to the plant pathway, cyanobacteria can completely decarboxylate glyoxylate to CO_2_ through an oxalate intermediate or bypass the plant pathway via a glycerate pathway (Eisenhut *et al*., 2008). The annotated genome of *P. tricornutum* lacks all these pathways except a truncated version of the plant‐like pathway (Kroth *et al*., 2008). Recently, an investigation into photorespiration in *T. pseudonana* found evidence for the full plant‐like pathway that converts mitochondrial serine to chloroplastic 2‐phosphoglycerate (2PG) (Davis *et al*., 2017). While homologs for enzymes in this pathway exist in *P. tricornutum*, they are poorly characterized and mitochondria‐targeted. Even with this pathway added to the model, SAL‐mediated pyruvate recycling is predicted. While energetically similar to 3PG and 2PG, pyruvate is a more versatile metabolite in *P. tricornutum* sitting at the intersection of mitochondrial anaplerotic reactions, C and amino acid metabolism. Additionally, unlike 2PG or 3PG, pyruvate can be integrated with chloroplast metabolism independent of the Calvin–Benson–Bassham (CBB) cycle. These results suggest several new hypotheses for alternate photorespiratory pathways in diatoms.

### Hypothesized cross‐compartment reductant shuttles

Model simulations, constrained with photophysiology data, detailed a diverse series of strategies that optimize energy use to avoid photo‐oxidative damage. Our results indicate that two dominant factors drive pathway selection: energy relocation ‘capacity’ (electrons transported per molecule) and oxygen consumption. The canonical pathways of mitochondrial C metabolism, ornithine‐glutamine shunt, photorespiration and BCAA degradation have defined energy yields based on reaction stoichiometry (Fig. S9). We combined these energy yields with the flux predictions at the experimental PAR values to determine C resource requirements and the fraction of linear electron flow that is transferred by these intracellular reductant shuttles (Fig. 8a,b).

Catabolism of CBB cycle outputs by the mitochondrial glycolytic pathway and TCA cycle has high‐energy dissipation capacity (*c*. 4.0 e^−^ per molecule; Fig. 8a,b) but does not consume oxygen outside of the mitochondrial ETC. The hypothesized ornithine‐glutamine shunt is a flexible pathway delivering C skeletons, reductant and N to the mitochondria. In our simulations, energy relocation capacity was dynamic, spanning from 1.3 to 3.7 e^−^ per molecule (Fig. 8a,b). When operating as a closed cycle, this pathway accrues zero mass loss (Fig. S9). Photorespiration has an energy relocation capacity of 1 e^−^ per molecule and the pathway consumes oxygen in both the Rubisco oxygenase and GOX reactions. Recent *in vitro* characterization of *P. tricornutum* mitochondrial GOX revealed that it can use multiple electron acceptors besides O_2_ (Schmitz *et al*., 2017). When we added this capability to the model, the majority of the flux continued to utilize O_2_ (data not shown). However, at high light, the model suggested a cycle interconverting glycolate and glyoxylate, providing an effective photoprotective mechanism by consuming NADH and O_2_ in the process. Still, the rate of photorespiration is dependent on O_2_ and CO_2_ concentrations proximal to Rubisco. These concentrations are not included in our simulations; thus, the high flux predicted may be an artifact of the model limitations. Computational methods such as CCM modeling (Hopkinson, 2014) or experimental approaches such as isotope‐labeled metabolic flux analysis are necessary to validate our predictions.

Branched‐chain amino acid synthesis and catabolism have the highest energy relocation capacity of all the predicted pathways; *c*. 9.3 e^−^ per molecule if oxidized to pyruvate (Fig. 8a,b). There is evidence that BCAA metabolism may also be involved in energy storage. Transcript‐level expression of BCAA catabolic enzymes in *P. tricornutum* were shown to be upregulated in the dark period and in response to iron limitation (Smith *et al*., 2016) and when adjusting to N limitation (Ge *et al*., 2014). The transcriptional regulatory network for *P. tricornutum* inferred from gene expression data showed coregulation between BCAA degradation and the TCA cycle, suggesting a link between BCAAs and central C metabolism (Smith *et al*., 2016; Levering *et al*., 2017). Dark‐period metabolic activity in *P. tricornutum* exceeded the energy provided by intracellular sugar and lipid stores (Jallet *et al*., 2016); temporal segregation of BCAA synthesis and catabolism between the day and night could explain this discrepancy. Additionally, temporal segregation would allow excess reductant to perform metabolic work as opposed to being dissipated through reductant‐mediated O_2_ consumption.

There is uncertainty in how BCAA catabolism interfaces with mitochondrial C metabolism (Pan *et al*., 2017). Canonically, valine catabolism connects with the TCA cycle through a set of three enzymes that convert propanoyl‐CoA to succinyl‐CoA. Previous investigations suggested the second step in this conversion is absent in *P. tricornutum* (Pan *et al*., 2017). Our GEM includes the proposed missing enzyme, methylmalonyl‐CoA epimerase, albeit with a low confidence annotation (Phatr3_J46728). Still, Phatr3_J46728 showed a similar diel gene expression profile with the upstream enzyme, propionyl‐CoA carboxylase, and the downstream enzyme, methylmalonyl‐CoA mutase (Smith *et al*., 2016) (Fig. S10).

The malate shuttle, an important chloroplast‐mitochondria metabolic link in higher plants (Kinoshita *et al*., 2011), has been hypothesized to function in *P. tricornutum* (Prihoda *et al*., 2012; Bailleul *et al*., 2015). The protein encoded by the *P. tricornutum* gene Phatr3_EG02645 shares domain homology with the Arabidopsis DIT1 2‐oxoglutarate‐malate exchanger. However, the gene model lacks an ER transit peptide signal and the diatom chloroplast targeting signal (Gruber *et al*., 2007). Thus, the localization and function of this putative transporter are unknown. Additionally, our GEM does not suggest the occurrence of any chloroplast‐localized enzymes in *P. tricornutum* that use malate as a substrate, which is in agreement with the idea that *P. tricornutum* does not encode a C4 pathway (Ewe *et al*., 2018).

However, DIT1 in higher plants also functions as a 2‐oxoglutarate/oxaloacetate exchanger. We tested this hypothetical functionality in *P. tricornutum* by adding the exchanger to our GEM and re‐running the simulations at low and high light. At high light, the predicted pathways were identical to the previous simulations. At low light, our simulations suggest this shuttle links chloroplast and mitochondria C metabolisms via a cytosolic ATP‐citrate synthase, replacing the amino acid transport and transaminase reactions previously predicted (Fig. S11; Dataset S1). Thus, our simulations suggest that if *P. tricornutum* contains a 2‐oxoglutarate/oxaloacetate exchanger, it could contribute to cross‐compartment metabolic coupling.

### Modeling assumptions and limitations

Like all models, our simulations include assumptions and limitations. The simulations did not include constraints on intracellular reaction fluxes, including transport. Thus, the relative flux through the intercompartment reductant shuttles could be regulated *in vivo* by enzyme capacity which is not currently modeled. Pools of reduced metabolites and futile metabolic cycles may also serve as energy‐dissipating mechanisms.

Photoacclimation strategies beyond pigment content and cellular absorption coefficients used in this study include thylakoid and light‐harvesting complex remodeling and energy dissipation upstream of the photosystem, such as NPQ. Quantitative constraints for thylakoid and light‐harvesting complex remodeling have yet to be developed, limiting their incorporation into GEMs. An accessible next step is to incorporate Chl fluorescence parameters, quantifying NPQ and nonradiative dissipation of excitation energy. These data could properly constrain the quantum efficiency of PSII in the model, increasing the agreement between MIMS data and model simulations. Nonradiative dissipation cannot be predicted by the GEM and must be fixed based on experimental approximations. We assumed the upper boundary for chlororespiration flux to be 10%, as Bailleul *et al*. (2015) measured similar light‐dependent oxygen consumption rates. We also assumed that up to 20% of absorbed photons could be lost as a result of NPQ, in line with observations in previous studies (Jakob *et al*., 2007). When we increased NPQ to 40%, similar intercompartment reductant shuttles were predicted but at higher PAR values (e.g. photorespiration wasn't observed until 300 μmol photons m^−2^ s^−1^; data not shown).

Although we reported flux values for the parsimonious flux solution, there are alternate pathways to those suggested by our results. Setting the flux through the ornithine‐glutamate shunt to zero resulted in additional flux being routed through the TCA cycle and no change to the observed growth rate (Dataset S1). Thus, the predicted pathways are hypotheses and require further validation. Isotope‐labeled metabolic flux analysis (Young *et al*., 2011) could provide experimental support for our predictions and constrain the magnitude of the fluxes.

### Conclusion

In this investigation, we incorporated photophysiology constraints with cellular composition dynamics in order to explore photoacclimation in a model marine diatom. These constraints resulted in accurate predictions of photoautotrophic growth, indicating that these are among the key dominant constraints on phototrophic metabolism. Our implementation of biomass composition dynamics characterized nonsteady‐state growth over a circadian cycle. Data‐dependent relaxation of the steady‐state assumption is an important extension of constraint‐based modeling and brings into scope a wide variety of phenotypes of interest to the phototrophic community.

Our simulations hypothesized intercompartment reductant shuttles used by *P. tricornutum* to relocate and consume excess light energy and support the hypothesis that photorespiration is an active photoprotection mechanism in diatoms. The hypothesized shuttles highlight how species‐specific metabolic innovations enable increased fitness to environmental factors, such as light availability. These shuttles may also serve as novel templates for engineering strategies aimed at increasing bioproducts from diatoms. The dramatic reorganization of cellular metabolism as a function of the light environment suggested by our simulations has design implications for metabolic engineering strategies to include heterologous pathway selection and bioprocess conditions. Our results highlight the value of systems‐level analysis and present new directions for exploring diatom physiology.

## Author contributions

ND and JTB planned, designed, and performed the research. ND conducted the physiological experiment. JTB performed GEM model simulation and flux analysis. JTB, YT and YM conducted the chloroplast volume measurement. ND, JTB and SRS analyzed the data. MAW performed the MIMS experiment and analyzed the data. ND, JTB, SRS, CLD, AEA, BGM, DJ, MAW, GP and BOP wrote and reviewed the manuscript. JTB and ND contributed equally to this work.

## Supporting information

Please note: Wiley Blackwell are not responsible for the content or functionality of any Supporting Information supplied by the authors. Any queries (other than missing material) should be directed to the *New Phytologist* Central Office.

**Dataset S1** Modeling files can be found at http://systemsbiology.ucsd.edu/Downloads/SupplementalData.
**Fig. S1** Biophysical constraints for simulation of *P. tricornutum* under a sinusoidal light regime.
**Fig. S2** Comparison of chloroplast volume determined from *z*‐stacked and 2D confocal microscopy images.
**Fig. S3** Sensitivity analysis of biophysical constraints for simulations of *P. tricornutum* under a sinusoidal light regime.
**Fig. S4** Sensitivity analysis of biomass components for simulations of *P. tricornutum* under a sinusoidal light regime.
**Fig. S5** Physiology metrics and simulation comparisons for *P. tricornutum* acclimated to four PAR values.
**Fig. S6** Photophysiology metrics for *P. tricornutum* acclimated to different light intensities.
**Fig. S7** Relative spectral irradiance of cool white fluorescence light for cultivation and LED light for P vs E measurements.
**Fig. S8** Coexpression connectivity between GOX_m and KOG annotated pathway clusters.
**Fig. S9** Energy relocation capacity of the predicted intercompartment reductant shuttles.
**Fig. S10** Gene expression profile of BCAA enzymes.
**Fig. S11** Hypothetical 2‐oxoglutarate shuttle in *P. tricornutum*.
**Methods S1** Cell cultivation, growth monitoring and physiology.
**Methods S2** Plastid volume determination.
**Methods S3** Photophysiology constraints.
**Methods S4** Photoautotrophic simulations of cellular growth.
**Methods S5** Membrane inlet mass spectrometry
**Table S1** Summary of changes in the genome‐scale model of *P. tricornutum*.
**Table S2** Dynamic biomass acclimation over the light period in *P. tricornutum* under different light regimes.
**Table S3** Chloroplast volume derived from confocal microscopy images of *P. tricornutum* acclimated to different light conditions.
**Table S4** Reconstructed carbon balance of the derived model biomass objective function.
**Table S5** Pigment profiles of *P. tricornutum* at different acclimated square‐wave light conditions.
**Table S6** Chloroplast resource allocation as a function of photoacclimated PAR.
**Table S7** Averages of membrane inlet mass spectrometry (MIMS) light‐dependent oxygen measurements.
**Table S8** Conversion of published lipid class concentrations (Abida *et al*., 2015) to mass values for incorporation into the GEM biomass objective function.
