# Supplementary material for: Cross‐compartment metabolic coupling enables flexible photoprotective mechanisms in the diatom Phaeodactylum tricornutum
Source: New Phytol. 2019 Feb 14;222(3):1364–79. doi: 10.1111/nph.15685 (PMC6594073; doi:10.1111/nph.15685)

***New Phytologist* Supporting Information**

Article title: Cross-compartment metabolic coupling enables flexible photoprotective mechanisms in the diatom *Phaeodactylum tricornutum*

Authors: Jared T. Broddrick*, Niu Du*, Sarah R. Smith, Yoshinori Tsuji, Denis Jallet, Maxwell A. Ware, Graham Peers, Yusuke Matsuda, Chris L. Dupont, B. Greg Mitchell, Bernhard O. Palsson, Andrew E. Allen

Article acceptance date: 20 December 2018

The following Supporting Information is available for this article:

Modeling files (Dataset S1) can be found at http://systemsbiology.ucsd.edu/Downloads/SupplementalData

Methods S1: Cell cultivation, growth monitoring and physiology. Marine diatom *Phaeodactylum tricornutum* purchased from Culture Collection of Algae and Protozoa (CCAP 1055/1 strain) were cultivated in 1 L vertical tubular bioreactors submerged in a temperature-controlled water tank at 20°C. Cool-white fluorescent lamps were installed horizontally at the back of the waterbath for illumination. A gradient of light intensities was established in the water tank by placing neutral density light filters of different attenuation levels in front of the lamps, with which we created homogenized light fields in 4 tubular bioreactors at the densities of 60, 120, 300 and 600 µmol photons m^{-2} s^{-1}. A 12 h : 12 h light : dark cycle was set by turning on and off the lamps with a timer. Diatom cultures inside the bioreactors were mixed by CO_2 enhanced air, the specific percentage of which was regulated by a Cole-Parmer gas flow controller to stabilize the culture pH at 8.1 $\pm$ 0.3. Before the experiment, all materials were sterilized to establish an axenic cultivation condition. Inoculations were carried out in f/2 medium 3 days before the first sampling point to allow sufficient time for acclimation, then the cells were diluted to 2-6x10^5 cells ml^{-1} and sampled over two light-dark cycles. Biomass accumulation was monitored with optical density at 750 nm (OD_{750}) during light periods. Analytical measurements were conducted at the end of the light period with harvested cells.

Chlorophylls and carotenoids were measured by HPLC. Aliquots of 50 and 100 mL were filtered on 2.5 cm Whatman GF/F filters and extracted with 3 mL of 90% acetone for 24 h. A reverse-phase stepwise gradient elution system with 3 HPLC grade solvents was employed: 90:10 MeOH; water (v/v). 94.6 MeOH:water (v/v), and 100% MeOH at a rate of 1 mL min^{-1}. The water in the first solvent contained an ion-pairing reagent (1) in concentrations of 1.5 g tetrabutylammonium and 0.96 g of ammonium acetate per 100 mL of water. Pigments were separated in a C-18 Econosphere column from Alltech with 5 µm particle size and dimensions of 4.3 mm x 25 cm. The column was calibrated with pure pigments obtained by injecting different volumes of an extract of known concentration. For this purpose, pigments were extracted from different unialgal cultures, isolated by thin-layer chromatography (2), dissolved in solvent, identified by its visible absorption spectrum, and quantified using the corresponding specific absorption coefficients. Pigment concentration was calculated by manually measuring the corresponding peak area.

For determination of the P vs E curve, samples were pelleted by centrifugation and re-suspended to a Chla concentration of approximately 3 µg/ml in f/2 medium. Cells were transferred into the glass cell of an ALGI^{TM} instrument for dissolved oxygen measurements. The mixing, light and temperature control for the measurement was achieved by the ALGI^{TM} that has been validated previously (3) (4). During measurement, samples were treated with alternative dark/light periods with 2 min interval and at increasing light intensity steps of 10, 20, 40, 80, 160, 320, 640, 1280 and 2500 µmol quanta m^{-2} s^{-1}. Measurement of O_2 recorded at 10 Hz during the dark/light periods for computing O_2 concentrations and subsequently the rate of change. Rates of oxygen evolution during the light periods were used to create the P vs. E curves. For the curve fitting we set the data to start at 0 so we could use the Platt et al. (5) function.

Variable fluorescence (Fv/Fm) determination. Fv/Fm was determined using a Chelsea Instruments FASTtracka FRR II fluorometer, which was programmed to deliver 100 saturation flashes per sequence with a flash duration of 1.1 µs at a 1 µs interval and the PMT gain set to auto-ranging mode. Upon sampling, the samples were diluted with culture media and inoculated into the induction chamber for 5 minutes of dark acclimation before measurement. The value of Fv/Fm was calculated using Fasttracka II software with the default settings.

Fatty Acid Methylester (FAME) Analysis. Phaeodactylum cells (15mL) were collected for FAME analysis on 25mm, 0.2µm GTTP filters (Millipore); the pellet was immediately resuspended into 2mL fresh ASW medium and stored at -80 °C. Upon analysis, lipids were transesterified and their FAME compositions and concentrations determined using gas chromatography with flame ionization detection (FID). In short, FAMEs were prepared and analyzed using methods derived from AOCS Ce 1b-89 and AOCS Ce 1-62, respectively. Dried samples in 4 mL glass vials were resuspended in 0.5 mL of 0.5M KOH in MeOH, 0.5mL Ottawa sand, and 80 μL of an internal standard mix containing C11:0 FFA/C13:0 TAG/C23:0 FAME. Samples, capped with PTFE-lined caps, were homogenized in a Geno/Grinder at 1200 rpm for 10 min, heated at 80°C for 30 min, then homogenized for an additional 5 min. 0.5 mL of 14% BF3 in MeOH was added to the samples, and after heating and homogenizing, 2mL of n-heptane followed by 0.5 mL of saturated NaCl were added. The samples were homogenized for a final time for 1.5 min, centrifuged at 1000 rpm for 3 min, and the upper phase sampled directly for GC-FID analysis.

Total protein analysis. Phaeodactylum cells (15mL) were collected for protein analysis on 25mm, 0.2µm GTTP filters (Millipore). The filter was flash frozen in liquid nitrogen and kept at -80°C. Pellets were thawed in lysis buffer (125 mM Tris-HCl, pH 6.8; 200 mM NaCl and 1 mM phenylmethanesulfonylfluoride). Cells were lysed at 4°C by sonication (Bioruptor; Diagenode, Denville, NH). Protein concentrations were determined by BCA assay with Htrf Flexstation 3.

Methods S2: Plastid volume determination. *P. tricornutum* UTEX 642 was cultured in TrueLine 25cm^2 vented culture flasks with approximately 15 mL of f/2 ASW at 20°C. Cultures were acclimated to 10, 150 and 600 µmol photons m^{-2} s^{-1} of light from

a white LED and the photon flux was determined with a LI-COR LI-250A light probe. Cultures were sampled in early-mid exponential phase, chlorophyll content determined, and imaged using a Leica TCS SP8 confocal microscope. Chlorophyll *a* concentration was determined by pelleting cells by centrifugation and extracting pigments with 90% MeOH/10% DMSO. Chlorophyll concentration was determined using the coefficients as reported in (6). For imaging,chlorophyll autofluorescence of representative cells was detected at 640-750nm following laser excitation at 552nm.

Modeling the chloroplast as two ellipsoids, we derived an equation (1) that allows for the approximation of chloroplast volume based on a single 2D image of chlorophyll auto-fluorescence, assuming the plane of the image approximately bisects the cell.

$$V_{lobe} = \frac{4}{3}\pi(L \times W \times \frac{1}{2}H) \quad [1]$$

V_{lobe} is the total volume of a chloroplast lobe, of which there are two in *P. tricorutum*. L is the semi-major axis of the lobe and W is the semi-minor axis. Consistent with the existing literature, we assumed the cell was a prolate sphere (7); thus, H is the thickness of the cell where thickness is equal to the width of the cell measured from the confocal bright field image. Dimensions were calculated in Fiji (8) using the straight-line tool and the image scale bar. Whole cell dimensions were determined on the bright field image and chloroplast dimensions were determined using the chlorophyll auto-fluorescence channel.

Chloroplast volume determined from z-stacked images was used to validate Equation (1). Using Fiji (8), each image of chlorophyll auto-fluorescence was split and the red channel isolated. The threshold was adjusted to remove pixels from out of focus sections of the image and the Analyze Particles function was used to return the image area in μm^2. The incremental change in the z-axis between each image (delta z) for the stack was used to generate the slice volume and the sum of all image slices was set as the total volume for comparison.

The final step required the conversion of chloroplast volume into lipid mass per cell. The lipid content of *P. tricornutum* was well characterized by Abida et. al. (9). Figure 4 of the paper reports quantitative values for each of the glycerolipids in nmol per $1x10^6$ cells. Lipids were converted into picogram per cell using the mean gram formula weight of the different side chains for a given lipid species represented in the genome-scale model's biomass function. The lipid classes were then separated into chloroplast lipids (ASQ, PG, SQDG, DGDG, MGDG and 1,2-DAG) and non-plastid membrane lipids (PC, PE, DGTA and PI). Using the regression equation of chloroplast size versus chlorophyll *a* (Fig.1a), the chloroplast volume of the cells in Abida et.al. was approximated using their reported irradiance value (100 µmol photons m^{-2} s^{-1}). From there the mass per cell allocated to chloroplast lipids and membrane lipids for each of the photoacclimated cultures was derived by taking the chloroplast volume at the experimental Chla concentration compared to that for the cells in Abida et. al and multiplying the resulting ratio by the lipid mass per cell (Table S8).

Methods S3: Photophysiology constraints. The biophysical constraints were based on an extension of our previous modeling of photoautotrophy in cyanobacteria (10). Briefly, using the spectral distribution of photon flux for the given light source at the experimental irradiance ($E_0(\lambda)$), the chlorophyll *a* specific spectral absorption coefficient (a^*_λ), and the biomass fraction of chlorophyll *a*, the photon uptake flux (E_a) was determined using the following equation:

$$E_a = \frac{mgChla}{gDW}\int_{400}^{700} E_0(\lambda)a^*_{ph}(\lambda)d\lambda \quad [2]$$

In vivo whole cell absorption was determined at 1 nm intervals using a dual beam spectrophotometer equipped with an integrating sphere. The chlorophyll-specific absorption coefficient was estimated using Equation (3), in which $a^*_{ph}(\lambda)$ is the chlorophyll *a* specific absorption coefficient, and $a_{ph}(\lambda)$ is the absorption coefficient. Both parameters are spectrally distributed.

$$a^*_{ph}(\lambda) = \frac{a_{ph}(\lambda)}{[Chla]} \quad [3]$$

To fully capture the wavelength specific light-pigment interactions, we switched from photosynthetically available radiance (PAR) to quantum flux (QF) (11), which describes the total absorbed photon flux, as the fundamental variable the oxygen evolution constraint:

$$QF = \int_{400}^{700} a^*_{ph}(\lambda) \times PAR \times E^{'}(\lambda)d\lambda \quad [4]$$

where $E^{'}(\lambda)$ is the fraction of total PAR at a given wavelength λ. The measured photosynthetic rates (oxygen evolution in this study) were then fitted to a Platt (5) equation for photosynthesis prediction (P), using quantum flux as the independent variable.

$$P = P_{max}(1 - e^{-\frac{\alpha \times QF}{P_{max}}})e^{-\frac{\beta \times QF}{P_{max}}} \quad [5]$$

P_{max} is the maximum photosynthetic rate, α and β are the parameters that describe the initial slope of the curve, and the photoinhibition (if present), respectively.

Methods S4: Photoautotrophic simulations of cellular growth. The *P. tricornutum* genome-scale model (GEM), *i*LB1025 (12), was updated with recent advances in diatom metabolic understanding (Table S1).

To model photoautotrophic growth under a sinusoidal light regime we first generated a new biomass objective function (BOF) based on published data (13). Biomass components reported in pg/cell were combined with the reported cell counts and total culture volume to arrive at pg per culture. The protein % carbon reported in Jallet et. al. was different than that in the model biomass (44% vs. 53% respectively); the model % carbon was used for all simulations and calculations.

Protein, structural carbohydrates and rate of storage compound accumulation was determined from the recently published biomass distribution during a full circadian period under a sinusoidal light regime(13). DNA and RNA were set to the values in the original *P. tricornutum* genome-scale model publication(12). Pigment mass per cell was experimentally determined for each of the photoacclimated cultures (See above in Methods S3). Non-chloroplast associated lipids were taken from the previously published values (9).

The biomass components at dawn (T=0) were the initial parameters for the model simulation. The T=0 values for protein, carbohydrates, TAG, chlorophyll *a* (Chla) and chlorophyll *c* were taken from the data in Jallet et. al. DNA and RNA were left at the same values as the original GEM publication (12). Plastid and membrane lipids were calculated as outlined in the main text based on the Chla concentration reported in Jallet et. al. at T=0. Pigments other than Chla were set to the levels measured in the 875 µmol photons m^{-2} s^{-1} culture used to approximate the missing biophysical constraints (see main text). Since the pigments were measured in the late light period, the ratio of [Chla] at dusk vs. dawn in the Jallet et. al. data was used to approximate the dawn pigment content. Storage carbon, divided between TAGs and a β-1,4-glucan molecule with a mass ratio of 3:1, was added to the BOF using a biomass metabolite, carbon_storage_c. The initial content of storage compounds was less than 1% based on the reported values. At this point a full BOF for the dawn time point was established.

The time variant biomass accumulation at T=3, 6, 9, 12 and 24 hours was determined in a similar manner. It was assumed the non-linear accumulation of storage carbon and chlorophyll were the only non-linear dynamics in the biomass accumulation. The biomass allocation coefficients for these two components were adjusted until the simulation results recapitulated the experimental values. From this the percentages used in the dynamic biomass accumulation were determined (Table S2).

For the sinusoidal light condition, the model was simulated under a 12 h : 12 h light : dark cycle, f/2 media, with 500 mL total culture volume and light from a white LED placed above the culture as reported (13) for the sinusoidal culture and 800 mL total culture volume and light from 360° for the photoacclimated cultures in the square-form, tubular bioreactors. Simulations used the updated version of the GEM, *i*LB1034. The media was set to f/2 components as in the original publication. The BOF was updated to the dawn time point determined above. The initial biomass content in mg cell dry weight was determined by summing the mass of all the individual components (10.19 mg DCW). The cross-sectional area of the photobioreactor used to calculate the photon flux was determined to be 22 cm^2 based on the reported culture height and volume.

Reaction constraints were set in a similar manner as in the original GEM publication to include constraints on cyclic electron flow, chloroplast water-water reactions and energetic coupling between the chloroplast and mitochondria as previously reported (14). Energetic coupling was set such that at least 15% of photosynthetically derived electrons were moved to the mitochondria.

Sinusoidal light regime. For the sinusodal light regime culture, the simulation period of 24 hours was divided into 20 minute time intervals. The biomass objective function was updated at the beginning of each interval in accordance with the derived dynamic biomass allocation (Table S2). To account for the dynamic biomass allocation at each time interval, a linear interpolation of the rate of change for each biomass component was used to update the BOF prior to each simulation. This new BOF was captured in a reaction named bof_c_accumulation_c, representing the carbon accumulation focus of the light period. The total photon flux was determined for each time interval by first parsing the pigment component of this BOF to determine the total Chla content of the culture at the time interval. The PAR for the simulation was calculated from the sinusoidal equation given in Jallet et. al. For a given 20 minute time interval, the available PAR was calculated from the middle of the time interval (e.g. for the time interval from 100-120 minutes, the PAR was calculated using t=110). The photon flux at each wavelength in the range 400-700 nm was determined using the relative spectral irradiance (Fig. S7), PAR and photobioreactor cross-sectional area (final units: µmol photons (time interval)$^{-1}$). The culture was subdivided into 50 vertical slices each containing 2% of the total biomass to calculate the effect of cell shading in the light column. The wavelength specific quantum flux was calculated for each slice according to Equations 3 and 4. The slice specific quantum flux was used to determine the slice specific oxygen evolution according to Equation 6. Photon absorption for the slice was tracked and used to adjust the input PAR for the next slice, effectively accounting for shading in the culture. Photon absorption and oxygen evolution were integrated over the culture height and these values were set as the upper and lower bound of the photon exchange reaction (EX_photon_e) and the upper bound of the oxygen exchange reaction (EX_o2_e). Non-growth associated maintenance (NGAM) was calculated from the experimental dark respiration rate. This value was set as the lower bound for an fictional alternate oxidase (NGAM), which forces a minimal amount of reductant mediated oxygen consumption consistent with the observed dark respiration rate.

The simulation was performed by maximizing the bof_c_accumulation_c reaction using the parsimonious FBA function (15) as implemented in COBRApy (16). The flux through this reaction is equal to the biomass accumulation in milligrams over the 20 minute time interval. This flux value was multiplied by the stoichiometric coefficients of the biomass components in the bof_c_accumulation_c reaction. For example, at T=0, the stoichiometric coefficient for protein biomass was 0.79, equal to the 79% of biomass going to proteins in the dynamic biomass table (Table S2). A bof_c_accumulation_c flux of 0.1 would result in a protein biomass accumulation of 0.079 mg over the time interval. Accumulation of all the individual biomass components was written to a file at the end of each time interval simulation. This recorded the simulated dynamic biomass accumulation as

well as parameterized the simulation for the subsequent time interval. This process was repeated for the duration of the light interval (T=0 to T=720 minutes) with the output of one interval parameterizing the next.

Dark period simulations were carried out in a similar fashion. A new BOF named bof_dark_c was constructed that included all biomass components except Chla since *P. tricornutum* lacks a light-independent protochlorophyllide oxidoreductase (17). We assumed the biomass ratios at T=0 were the target ratios for cell division. Thus, the biomass component ratios at T=720 were parsed and compared with the desired ratios. The bof_dark_c stoichiometric coefficients were constructed to balance the biomass component ratios to the T=0 values. Upon achieving the target ratios, biomass was allowed to accumulate at the target ratios for the remainder of the simulation.

Dark period specific constraints included setting the bounds for photon flux, RubisCO and carbon storage to 0. The accumulated carbon storage from the light period was made available to the model for growth. The values for accumulated β-1,4-glucan and TAG (captured as a representative metabolite TAG(16:1/16:1/16:0)) were allowed to decrease linearly across the dark period as seen in Fig. 5f. The BOF reaction was optimized and biomass component accumulation as well as carbon storage compound consumption were tracked as in the light period. The final output was a time-course biomass accumulation table for each biomass component seen in Fig. 2.

Block-light regime at four irradiances. To model photoautotrophic growth at 60, 120, 300 and 600 µmol photons m^{-2} s^{-1} of PAR, we generated unique BOFs for each light intensity. The BOF generated for sinusoidal growth above served as the basis with the relative percent of pigments updated based on the experimental results (Table S5) and the photosynthetic lipids adjusted based on the Chla concentration (Main Text Fig. 1a). We used the time course data for the high and low light conditions to adjust the dynamic biomass allocation; with values for 120 and 300 µmol photons m^{-2} s^{-1} determined by interpolation of the 60 and 600 µmol photons m^{-2} s^{-1} coefficients (Table S2).

Simulations used the updated version of the GEM, *i*LB1034. The media was set to f/2 components as in the original publication. The BOF was updated to the dawn time points determined above. The initial biomass content in mg cell dry weight for each culture was determined by taking the T=0 cell count, multiplying by the pg POC cell^{-1} and then dividing by the carbon fraction of total biomass as reported in the BOF.

The T=0 pg DW cell^{-1} were: 60 µmol photons m^{-2} s^{-1}: 18 pg DW cell^{-1}, 20 pg DW cell^{-1}, 300 µmol photons m^{-2} s^{-1}: 22 pg DW cell^{-1}, 600 µmol photons m^{-2} s^{-1}: 23 pg DW cell^{-1}; with the values at 120 and 300 µmol photons m^{-2} s^{-1} inferred from the experimental values at 60 and 600 µmol photons m^{-2} s^{-1}.

This resulted in the following initial mgDWs used for the simulation: 60 µmol photons m^{-2} s^{-1}: 3.4 and 4.9 mg, 120 µmol photons m^{-2} s^{-1}: 7.5 and 16.4 mg, 300 µmol photons m^{-2} s^{-1}: 13.6, 41.1 and 15.0 mg, 600 µmol photons m^{-2} s^{-1}: 15.8, 15.5 and 45.2 mg.

The cross-sectional area of the photobioreactor used to calculate the photon flux was determined to be 533 cm^2 based on the culture height of 28.3 cm and volume of 800 mL. Reaction constraints were the same as in the sinusoidal simulation above. The simulation was carried out in the same manner as the sinusoidal conditions. The BOF, PAR value, maximum oxygen evolution and Chla specific absorption coefficient were specific for the experimental condition. Only the light period was simulated (T=0 to 720 minutes).

Photoacclimated cultures' physiological data was reported in mass per cell. Since the model outputs are in units of mass, the per cell normalized values needed to be converted into total mass for comparison. Experimentally determined OD to cell count conversions are only applicable early in the light period as OD increases along with biomass but cell division is somewhat light synchronized to the end of the light period (13). We used the time course data for the high and low light conditions to adjust the dynamic biomass allocation; with values for 120 and 300 µmol photons m^{-2} s^{-1} determined by interpolation of the 60 and 600 µmol photons m^{-2} s^{-1} to derive the cell counts. The error in cell count approximation was combined with the standard deviation of the experimental measurements to establish the total standard deviation reported (Main Text). The simulation values used for comparison with the experimental physiology data were the simulation outputs at T=9 hrs; consistent with the sampling time point.

Methods S5: Membrane inlet mass spectrometry. *P. tricornutum* CCAP 1055/1 were cultivated in 1 L Roux flasks and maintained at 20C. Computer controlled white light LEDs were used for illumination and adjusted to achieve 60, 120, 300 and 600 µmol photons m^{-2} s^{-1}, under a 12 h : 12 h light : dark cycle. Cultures were bubbled with atmospheric air.

A quad mass spectrometer (Pfieffer PrismaPlus QMS200; PTM28612) was used to measure dissolved oxygen concentrations. All experiments were performed using a custom designed 2 ml glass cuvette equipped with a carbon stopper. Cells were collected according to Methods S1 and supplemented with 20 mM HEPES buffer pH 7.5 and 5 mM sodium bicarbonate (Jallet et al., 2016). Cells were resuspended to 3 µg chla final concentration (2.5 µg chla/ml). These cells were flushed with N_2 until $^{16}O_2$ reached approximately half atmospheric levels. 2 ml of sample was transferred to a custom quartz oval cuvette (1cm maximum pathlength), with the stopper being lowered to the sample surface. $^{18}O_2$ (Cat # 490474 ALDRICH) was then injected through the stopper. Once $^{18}O_2$ reached the same concentration as $^{16}O_2$, the stopper was lowered to a final sample volume of 1.2 ml. Data collection was carried out according to Jallet et al., 2016, with cells were kept for 5 min at 0 µmol photons m^{-2} to measure dark respiration rates; 15 min, measuring light corresponding to $\tilde{0}.2$ V to relax NPQ; and 5 min of white actinic light (60, 120, 300 or 600 µmol photons m^{-2}, (see Fig.2a (18) for spectral distribution). Samples were maintained at 20C throughout the experiment and kept constantly stirring. Rates of oxygen consumption and evolution were calculated once a steady state was reached during each illumination step (typically over 2-3 min). Oxygen evolution and consumption rates were calculated according to the equations developed by Beckmann et al., 2009 (19).

O_2 consumption $= \Delta^{18}O_2\ (1 + [^{16}O_2] / [^{18}O_2])$

O_2 evolution = $\Delta^{18}O_2$ - $\Delta^{16}O_2$ ($[^{16}O_2]$ / $[^{18}O_2]$)

Where $[^{16}O_2]$ and $[^{18}O_2]$ reflect the relative concentrations of $^{16}O_2$ and $^{18}O_2$ once a steady state was reached during each illumination step. Net photosynthesis rates were calculated according to total oxygen ($^{16}O_2$ + $^{18}O_2$) changes during each illumination step. To account for abiotic gas consumption and changes in pressure, argon concentrations were used to normalize oxygen concentrations. Oxygen concentrations were calibrated to air saturated F/2 media and following the addition of sodium dithionite (zero O_2).

1. Mantoura R, Llewellyn C (1983) The rapid determination of algal chlorophyll and carotenoid pigments and their breakdown products in natural waters by reverse-phase high-performance liquid chromatography. *Analytica Chimica Acta* 151:297–314.
2. Guillard RRL, Lorenzen CJ (1972) YELLOW-GREEN ALGAE WITH CHLOROPHYLLIDE c2. *Journal of Phycology* 8(1):10–14.
3. Meuser JE, Boyd ES, Ananyev G, Karns D, Radakovits R, Murthy UMN, Ghirardi ML, Dismukes GC, Peters JW,, Posewitz MC (2011) Evolutionary significance of an algal gene encoding an [FeFe]-hydrogenase with f-domain homology and hydrogenase activity in chlorella variabilis NC64a. *Planta* 234(4):829–843.
4. Noone S, Ratcliff K, Davis R, Subramanian V, Meuser J, Posewitz MC, King PW,, Ghirardi ML (2017) Expression of a clostridial [FeFe]-hydrogenase in chlamydomonas reinhardtii prolongs photo-production of hydrogen from water splitting. *Algal Research* 22:116–121.
5. Platt T, Gallegos CL,, Harrison WG (1980) Photoinhibition of photosynthesis in natural assemblages of marine phytoplankton. *Journal of Marine Research* 38:687–701.
6. Ritchie RJ (2008) Universal chlorophyll equations for estimating chlorophylls a, b, c, and d and total chlorophylls in natural assemblages of photosynthetic organisms using acetone, methanol, or ethanol solvents. *Photosynthetica* 46(1):115–126.
7. Hopkinson BM, Dupont CL, Allen AE,, Morel FMM (2011) Efficiency of the CO2-concentrating mechanism of diatoms. *Proceedings of the National Academy of Sciences* 108(10):3830–3837.
8. Schindelin J, Arganda-Carreras I, Frise E, Kaynig V, Longair M, Pietzsch T, Preibisch S, Rueden C, Saalfeld S, Schmid B,, et al. (2012) Fiji: an open-source platform for biological-image analysis. *Nature Methods* 9(7):676–682.
9. Abida H, Dolch LJ, Meï C, Villanova V, Conte M, Block MA, Finazzi G, Bastien O, Tirichine L, Bowler C,, et al. (2014) Membrane glycerolipid remodeling triggered by nitrogen and phosphorus starvation inPhaeodactylum tricornutum. *Plant Physiology* 167(1):118–136.
10. Broddrick JT, Rubin BE, Welkie DG, Du N, Mih N, Diamond S, Lee JJ, Golden SS,, Palsson BO (2016) Unique attributes of cyanobacterial metabolism revealed by improved genome-scale metabolic modeling and essential gene analysis. *Proceedings of the National Academy of Sciences* 113(51):E8344–E8353.
11. Morel A (1978) Available, usable, and stored radiant energy in relation to marine photosynthesis. *Deep Sea Research* 25(8):673–688.
12. Levering J, Broddrick J, Dupont CL, Peers G, Beeri K, Mayers J, Gallina AA, Allen AE, Palsson BO,, Zengler K (2016) Genome-scale model reveals metabolic basis of biomass partitioning in a model diatom. *PLOS ONE* 11(5):e0155038.
13. Jallet D, Caballero MA, Gallina AA, Youngblood M,, Peers G (2016) Photosynthetic physiology and biomass partitioning in the model diatom phaeodactylum tricornutum grown in a sinusoidal light regime. *Algal Research* 18:51–60.
14. Bailleul B, Berne N, Murik O, Petroutsos D, Prihoda J, Tanaka A, Villanova V, Bligny R, Flori S, Falconet D,, et al. (2015) Energetic coupling between plastids and mitochondria drives CO2 assimilation in diatoms. *Nature* 524(7565):366–369.
15. Lewis NE, Hixson KK, Conrad TM, Lerman JA, Charusanti P, Polpitiya AD, Adkins JN, Schramm G, Purvine SO, Lopez-Ferrer D,, et al. (2010) Omic data from evolved e. coli are consistent with computed optimal growth from genome-scale models. *Molecular Systems Biology* 6:390.
16. Ebrahim A, Lerman JA, Palsson BO,, Hyduke DR (2013) COBRApy: COnstraints-based reconstruction and analysis for python. *BMC Systems Biology* 7(1):74.
17. Hunsperger HM, Ford CJ, Miller JS,, Cattolico RA (2016) Differential regulation of duplicate light-dependent protochlorophyllide oxidoreductases in the diatom phaeodactylum tricornutum. *PLOS ONE* 11(7):e0158614.
18. Lucker BF, Hall CC, Zegarac R,, Kramer DM (2014) The environmental photobioreactor (ePBR): An algal culturing platform for simulating dynamic natural environments. *Algal Research* 6:242–249.
19. Beckmann K, Messinger J, Badger MR, Wydrzynski T,, Hillier W (2009) On-line mass spectrometry: membrane inlet sampling. *Photosynthesis Research* 102(2-3):511–522.
20. Smith SR, Gillard JTF, Kustka AB, McCrow JP, Badger JH, Zheng H, New AM, Dupont CL, Obata T, Fernie AR,, et al. (2016) Transcriptional orchestration of the global cellular response of a model pennate diatom to diel light cycling under iron limitation. *PLOS Genetics* 12(12):e1006490.
21. Ewe D, Tachibana M, Kikutani S, Gruber A, Bártulos CR, Konert G, Kaplan A, Matsuda Y,, Kroth PG (2018) The intracellular distribution of inorganic carbon fixing enzymes does not support the presence of a c4 pathway in the diatom phaeodactylum tricornutum. *Photosynthesis Research* 137:263–280.

Table S1. Summary of changes in the genome-scale model of *P. tricornutum*. Reaction names are from *i*LB1025([12]). Changes are captured in *i*LB1034 included in this work. GPR: gene-protein-reaction.

Reaction	Change	Notes
PGTPt_m	Deleted	Transporter most likely substrate is G3P not 3PG.
PGTPti_h	Deleted	Transporter most likely substrate is G3P not 3PG.
GLUDC_m	Deleted	No GPR.
DHAD_4_m	Deleted	Chloroplast targeted based on fluorescent tagging.
4MOPt_h	Deleted	No evidence for transporter.
4MOPt_m	Deleted	No evidence for transporter.
3MOPt_h	Deleted	No evidence for transporter.
3MOPt_m	Deleted	No evidence for transporter.
Ru5PPi_th	Deleted	No evidence for transporter.
Xu5PPi_th	Deleted	No evidence for transporter.
6PGTt_h	Deleted	No evidence for transporter.
AKGMALt_h	Deleted	No evidence for transporter.
MALOAAt_h	Deleted	No evidence for transporter.
MALICITt_m	Deleted	No evidence for transport of this substrate.
AKGICITt_m	Deleted	No evidence for transport of this substrate.
OAAICITt_m	Deleted	No evidence for transport of this substrate.
EDD_m	Moved to plastid (EDD_h)	Chloroplast targeted based on fluorescent tagging.
ARG_c	Moved to mito (ARG_m)	Based on recent literature (20).
ARGt_m	Added	Required based on arginase localization to the mitochondria.
SBP_h	GPR	Added Phatr3_EG02409 as a isozyme based on homology.
ENO_m	GPR	Added Phatr3_Jdraft1192 as a isozyme based on homology.
GDR_NADP_m	GPR	Changed to Phatr3_J49167.
GLYTA_c	Changed to GLYTA_m	Reaction name error in model.
SPT_m	Added	Based on homology.
HYPRRx_m	Added	Based on homology.
GLYCK2_m	Added	Based on homology.
ASPGLU2_m	Added	Based on homology.
ASPTA_h	Edited	Phatr3_J23059 added Based on homology.
ME_x	Deleted	Based on recent literature (21).

Table S2. Dynamic biomass acclimation over the light period in *P. tricornutum* under different light regimes. Values indicate the percent of fixed resources allocated to that particular biomass component at the indicated time point, not the actual biomass composition. These percentages were determined in this study based on published, experimental values for the sinusoidal regime (13). For the block-light regime, values were determined by updating the sinusoidal dynamics with experimental values specific to a block-light regime. Struct: structural carbohydrates, Storage Carbon: chrysolaminarin and triacylglycerols (TAG), Pro: Protein, Pigm: pigments. Plastid lipids and membrane lipids are defined in Table S8.

Dynamic biomass allocation

Sinusoidal light regime (Jallet et. al. 2016)

Time	Total	Struct	Storage Carbon	Pro	Pigm	DNA	RNA	Plastid Lipids	Mem. Lipids
0	100	6.99	0.73	79.7	1.05	0.36	3.08	4.52	3.54
3	100	6.23	10.95	70.98	1.56	0.32	2.74	4.02	3.15
6	100	5.08	26.06	57.92	2.55	0.26	2.24	3.28	2.57
9	100	4.1	39.03	46.69	3.42	0.21	1.8	2.64	2.07
12	100	6.27	4.59	71.45	7.34	0.33	2.76	4.05	3.17

Block-light regimes (this study)

60 µE acclimation

Time	Total	Struct	Storage Carbon	Pro	Pigm	DNA	RNA	Plastid Lipids	Mem. Lipids
0	100	9.42	0.5	72.92	1.09	0.21	4.15	9.19	2.47
3	100	8.48	9.43	65.65	1.97	0.19	3.73	8.27	2.23
6	100	7.38	16.42	57.15	6.45	0.16	3.25	7.2	1.94
9	100	5.55	33.94	42.95	8.09	0.12	2.44	5.41	1.46
12	100	6.54	29.07	50.58	2.66	0.14	2.88	6.37	1.71

120 µE acclimation

Time	Total	Struct	Storage Carbon	Pro	Pigm	DNA	RNA	Plastid Lipids	Mem. Lipids
0	100	8.76	0.47	75.3	1.91	0.19	3.86	7.26	2.22
3	100	7.56	13.56	64.98	2.2	0.16	3.33	6.26	1.91
6	100	6.41	22.99	55.08	5.6	0.14	2.82	5.31	1.62
9	100	5.67	30.55	48.79	6.2	0.12	2.5	4.7	1.44
12	100	6.28	26.31	54.02	3.66	0.13	2.76	5.21	1.59

300 µE acclimation

Time	Total	Struct	Storage Carbon	Pro	Pigm	DNA	RNA	Plastid Lipids	Mem. Lipids
0	100	7.25	4.97	77.77	1.39	0.16	3.19	3.49	1.75
3	100	5.78	23.81	62.05	1.48	0.13	2.54	2.78	1.39
6	100	5.08	31.39	54.54	2.93	0.11	2.24	2.44	1.23
9	100	5.63	23.21	60.48	3.97	0.12	2.48	2.71	1.36
12	100	6.18	16.97	66.33	3.17	0.14	2.72	2.97	1.49

600 µE acclimation

Time	Total	Struct	Storage Carbon	Pro	Pigm	DNA	RNA	Plastid Lipids	Mem. Lipids
0	100	22.59	4.92	63.09	1.41	0.14	3.42	2.78	1.6
3	100	18.13	23.7	50.63	1.13	0.11	2.75	2.23	1.28
6	100	14.99	35.94	41.88	1.87	0.09	2.27	1.85	1.06
9	100	19.44	16.94	54.31	2.42	0.12	2.95	2.4	1.38
12	100	21.32	9.29	59.53	2.32	0.13	3.23	2.63	1.51

Table S3. Chloroplast volume determination. Chloroplast volume was derived from confocal microscopy images of *P. tricornutum* cells acclimated to different light conditions using equation (1). Light level is in μmol photons m^{-2} s^{-1}, length and width in μm and volume in $μm^3$.

		Whole cell dimensions		Plastid Lobe 1		Plastid Lobe 2		Plastid Lobe 1	Plastid Lobe 2	Total	Mean (Std Dev)
Cell #	Light level	Length	Width	Length	Width	Length	Width	Vol	Vol	Vol	Vol
1	15	26.7	3.5	4.4	2.9	4.9	2.4	23.4	21.6	44.9	
2	15	28.0	3.5	4.8	2.6	5.4	2.3	22.9	22.8	45.6	
3	15	26.0	3.3	3.5	2.3	3.6	2.3	13.9	14.3	28.2	40.5 (7.5)
4	15	25.0	3.5	3.9	2.4	4.0	2.5	17.2	18.3	35.5	
5	15	31.0	3.7	4.6	2.7	5.0	2.5	24.1	24.2	48.3	
6	150	23.8	3.8	6.5	1.4	3.4	1.0	18.1	6.8	24.9	
7	150	32.0	3.6	6.7	0.9	6.1	1.4	11.4	16.1	27.5	
8	150	24.0	3.3	3.4	2.3	2.1	2.1	13.5	7.6	21.1	23.9 (2.0)
9	150	25.2	3.1	4.4	1.6	3.1	2.6	11.4	13.1	24.5	
10	150	23.5	3.6	4.9	1.2	4.4	1.4	11.1	11.6	22.7	
11	150	23.7	3.2	5.4	1.5	4.3	1.3	13.6	9.4	22.9	
12	600	29.0	2.7	2.7	1.1	3.0	1.3	4.2	5.5	9.7	
13	600	25.0	2.9	2.9	1.5	2.0	1.0	6.6	3.0	9.6	
14	600	23.0	3.1	2.5	1.9	2.0	1.7	7.7	5.5	13.2	11.6 (2.5)
15	600	26.0	3.6	2.5	1.6	1.9	2.3	7.5	8.2	15.8	
16	600	27.0	2.8	3.3	0.8	2.3	1.7	3.9	5.7	9.6	

Table S4. Reconstructed carbon balance of the derived GEM biomass objective function over the light period for *P. tricornutum* cultured under a sinusoidal light regime compared to published total organic carbon (TOC) values (13). Carbohydrates, triacylglycerols (TAG), protein and the pigments chlorophyll *a* and *c* were reported in Jallet et. al. (2016). The pigments fucoxanthin, diatoxanthin, diadinoxanthin and beta-carotene were taken from a culture in this study (Fig. S1). DNA and RNA were taken from the original GEM of *P. tricornutum* (12). Plastid and membrane lipids were determined from the chlorophyll *a* content per cell (Fig. 1b) and literature values (9) respectively.

Reconstructed carbon balance

Sinusoidal light regime (pg C per cell)

Time	Structural carbs	Chrysolaminarin	TAG	Protein	Pigments	DNA	RNA	Plastid lipids	Non-plastid lipids	Total (model biomass)	TOC	Within experimental SD?
0	0.31	0.01	0.03	3.50	0.27	0.014	0.11	0.30	0.24	4.78	4.18±1.25	Yes
3	0.30	0.01	0.12	3.94	0.29	0.015	0.12	0.32	0.25	5.37	6.16±1.95	Yes
6	0.33	0.19	0.50	4.51	0.32	0.015	0.15	0.36	0.25	6.64	7.38±1.12	Yes
9	0.37	0.38	0.77	4.98	0.39	0.017	0.21	0.44	0.29	7.86	9.77±0.28	**No**
12	0.45	0.56	0.65	4.50	0.43	0.020	0.27	0.48	0.35	7.73	9.89±1.61	**No**
24	0.29	0.01	0.02	3.65	0.32	0.014	0.28	0.36	0.25	5.21	5.72±0.92	Yes

Table S5. Pigment profiles of *P. tricornutum* at different acclimated light conditions. Chl: Chlorophyll; C: Carbon; Fx: Fucoxanthin; Xan: Xanthophyll pigments (Diatoxanthin + Diadinoxanthin); β-car: Beta-carotene. Values are from a single biological replicate.

PAR	Chl*a* (pg $cell^{-1}$)	C:Chl*a* (g g^{-1})	Chl*c*:Chl*a* (g g^{-1})	Fx:Chl*a* (g g^{-1})	Xan:Chl*a* (g g^{-1})	β-car:Chl*a* (g g^{-1})
60	0.85	14.5	0.08	0.52	0.10	0.03
120	0.53	22.0	0.10	0.72	0.04	0.03
300	0.37	31.0	0.09	0.72	0.16	0.05
600	0.24	44.1	0.10	0.82	0.25	0.06

Table S6. Predicted chloroplast resource allocation as a function of photoacclimated PAR. Pigment values were determined experimentally and the resulting Chla content was used to approximate the chloroplast volume (Fig. 1a). This volume was converted to chloroplast lipid content using the data reported in (9) (Table S8). *Culture light intensity (µmol photons m^{-2} s^{-1}). The specific intensity of light is based on the averaged measurements at different locations of the bioreactor.

Chloroplast associated biomass components			
PAR*	Pigments (%biomass)	Lipids (%biomass)	Total (%biomass)
60	5.2	8.8	14.0
120	6.1	6.9	13.0
300	4.7	3.5	8.2
600	3.6	2.8	6.4

Table S7. Averages of membrane inlet mass spectrometry (MIMS) light dependent oxygen measurements. LED white lights were used for illumination. ± values represent standard deviations from biological triplicates, except for the 300 µmol photons m-2 s-1 condition which represents biological duplicates.

	Average gross oxygen evolution (µmol O_2 L^{-1} s^{-1} µg Chl a^{-1})	Average gross oxygen consumption (µmol O_2 L^{-1} s^{-1} µg Chl a^{-1})	Average net photosynthesis (µmol O_2 L^{-1} s^{-1} µg Chl a^{-1})	Average percentage of electron flow diverted to oxygen consumption (%)
600 µmol photons m^{-2} s^{-1}	0.176 ± 0.007	-0.043 ± 0.005	0.144 ± 0.012	20 ± 2
300 µmol photons m^{-2} s^{-1}	0.070 ± 0.016	-0.016 ± 0.012	0.065 ± 0.036	18 ± 15
120 µmol photons m^{-2} s^{-1}	0.082 ± 0.028	-0.040 ± 0.022	0.050 ± 0.003	31 ± 5
60 µmol photons m^{-2} s^{-1}	0.038 ± 0.005	-0.021 ± 0.005	0.029 ± 0.003	35 ± 5

Table S8. Conversion of published lipid class concentrations (9) to mass values for incorporation into the GEM biomass objective function. ASQ: acylsulfoquinovosyldiacylglycerol, PG: phosphatidylglycerol, SQDG: sulfoquinovosyldiacylglycerol, DGDG: digalactosyldiacylglycerol, MGDG: monogalactosyldiacylglycerol, DAG: diacylglycerol, PC: phosphatidylcholine, PE: , DGTA: diacylglyceryl-hydroxymethyl-N,N,N-trimethyl-β-alanine, PI: phosphatidylinositol, GFW: gram formula weight.

	Lipid class	nmol/10^6 cells	Mean GFW	pg/cell
Plastid lipids	ASQ	0.02	1124.57	0.02
	PG	0.15	731.96	0.11
	SQDG	0.42	792.10	0.33
	DGDG	0.15	889.16	0.13
	MGDG	0.81	730.99	0.59
	DAG	0.04	561.86	0.02
Membrane lipids	PC	0.33	727.99	0.24
	PE	0.03	686.93	0.02
	DGTA	0.08	804.15	0.06
	PI	0.05	808.01	0.04

Fig. S1. Biophysical constraints for simulation of *P. tricornutum* under a sinusoidal light regime. (a) Chla specific absorption coefficient for *P. tricornutum* acclimated to 875 µmol photons m^{-2} s^{-1} determined in this study and used to parameterize the sinusoidal light simulation. (b) Adjustment of the Chla specific absorption coefficient determined in panel (a) to recapitulate the reported light loss along the path length of the photobioreactor as reported in (13). (c) Pigment content determined in this study by HPLC analysis for *P. tricornutum* acclimated to 875 µmol photons m^{-2} s^{-1} used to parameterize the sinusoidal light simulation.

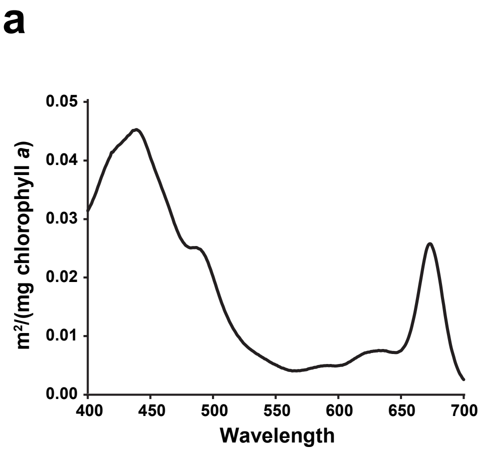

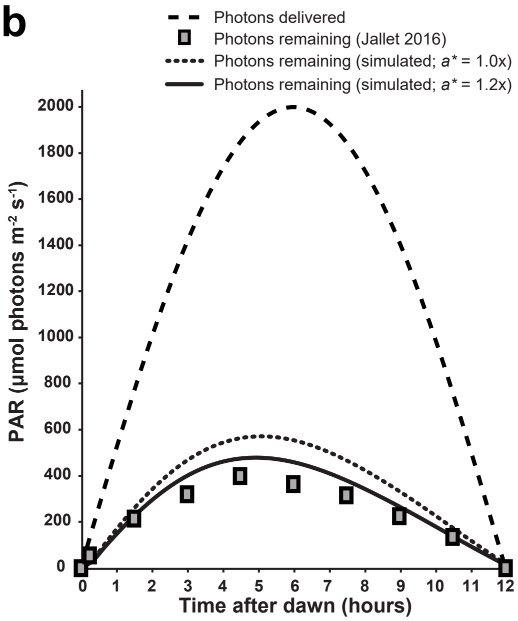

c

Pigment content

	chl *a*	chl *c2*	chl *c1*	fuco	diato	diadino	β-car
pg/cell	0.284	0.029	0.013	0.126	0.003	0.057	0.012

Fig. S2. Comparison of chloroplast volume determined from z-stacked and 2D confocal microscopy images.

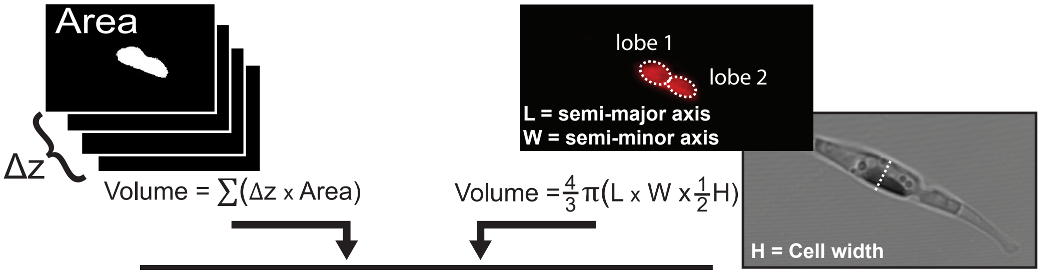

Irradiance (µE)	z-stack (µm³)	2D image (µm³)	%Difference
15	22.6	20.5	-10.2
15	46.8	45.1	-3.7
150	26.1	26.1	-0.2
600	7.1	6.5	-8.7
600	11.7	11.1	-5.4

Fig. S3. Sensitivity analysis of biophysical constraints for simulations of *P. tricornutum* under a sinusoidal light regime. Parameters were randomly perturbed away from their initial value by $\pm$0-20% for 1000 independent simulations.

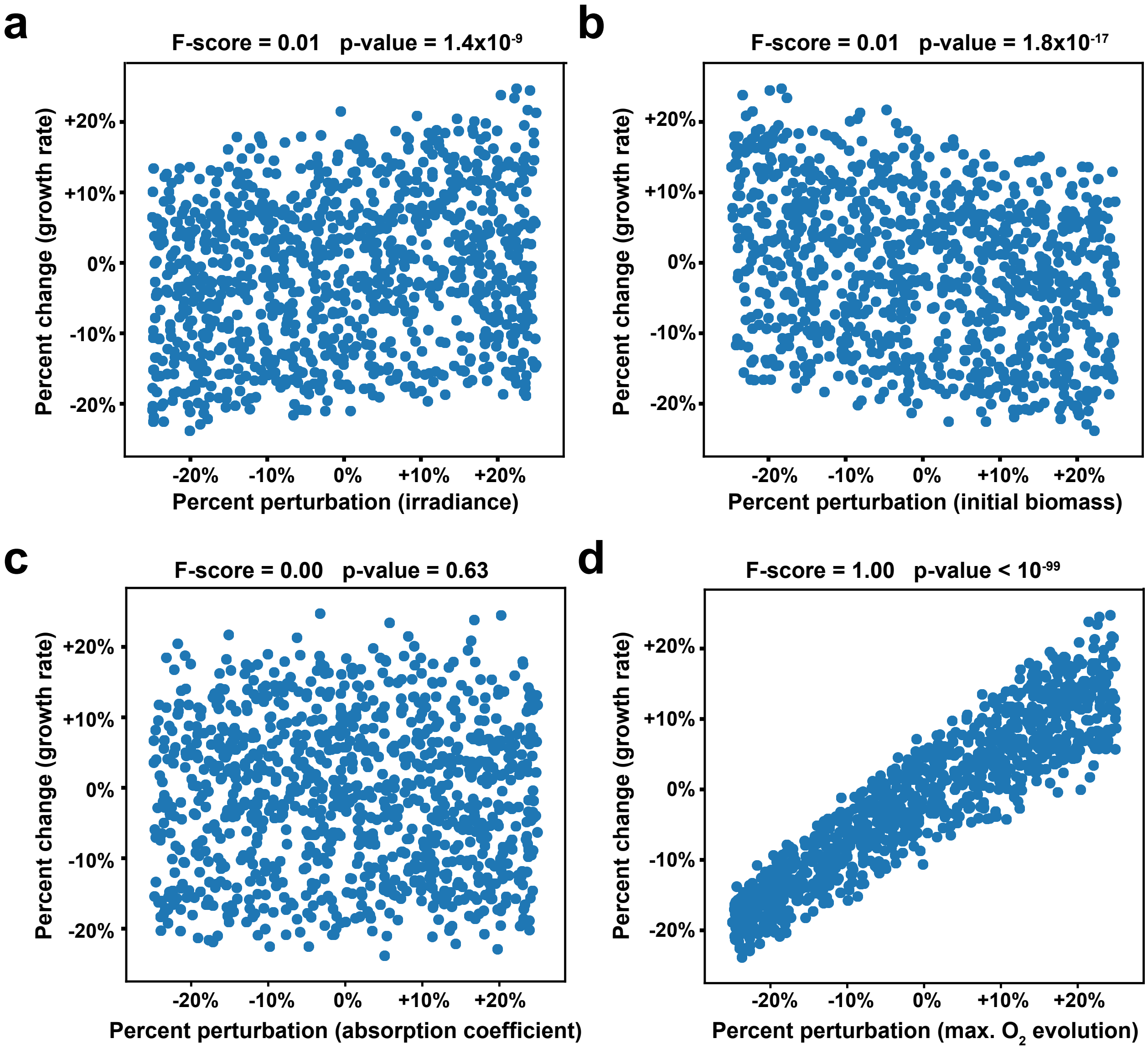

Fig. S4. Sensitivity analysis of biomass components for simulations of *P. tricornutum* under a sinusoidal light regime. Parameters were randomly perturbed away from their initial value by $\pm$0-30% for 1000 independent simulations.

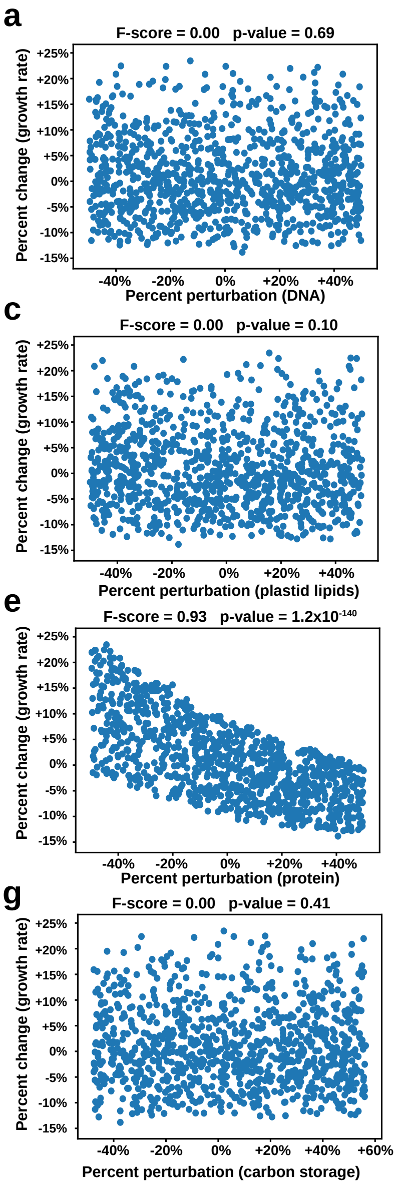

Fig. S5. Physiology metrics and simulation comparisons for *P. tricornutum* acclimated to four PAR values. (a) OD750 based growth rate. (b) POC, PN and Fv/Fm values. Error bars are the standard deviation of two technical replicates. (c) Comparison of experimental and simulated FAME accumulation dynamics in *P. tricornutum* acclimated to low (60μE) and high (600μE) light.(d) Comparison of experimental and simulated Chla accumulation dynamics in *P. tricornutum* acclimated to low (60μE) and high (600μE) light.

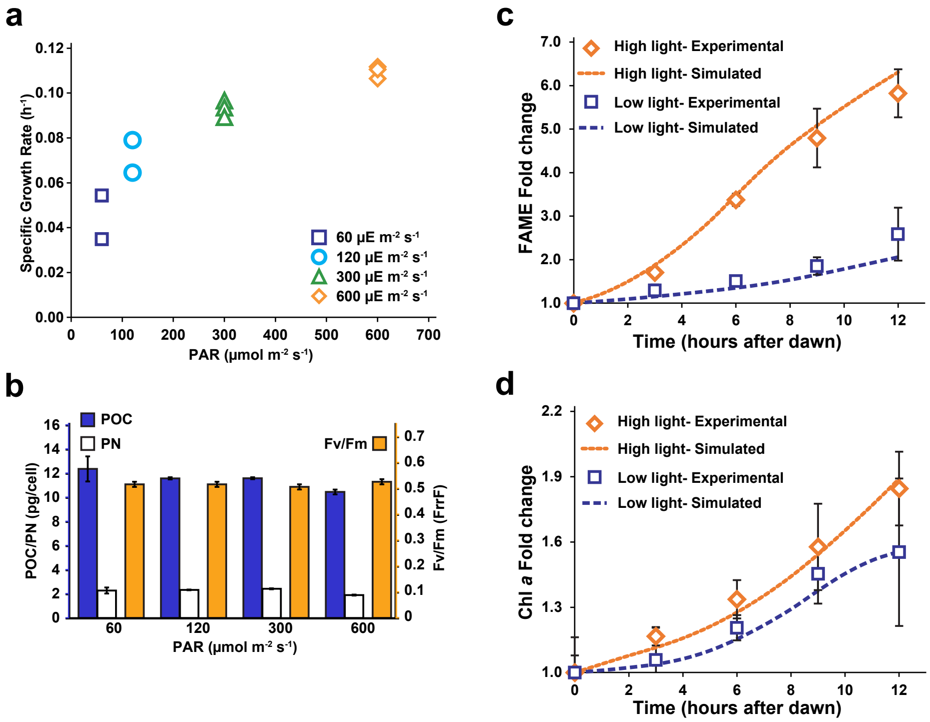

Fig. S6. Photophysiology metrics for *P. tricornutum* acclimated to different light intensities. (a) Chlorophyll *a* specific absorption coefficients determined in this study and used to parameterize the photon uptake rate in the genome-scale model simulations. Acclimated irradiance is indicated by color. (b) Net oxygen evolution light-response curve (P vs. I) of *P. tricornutum* samples acclimated to different PAR levels. Acclimated irradiance is indicated by color and shape.

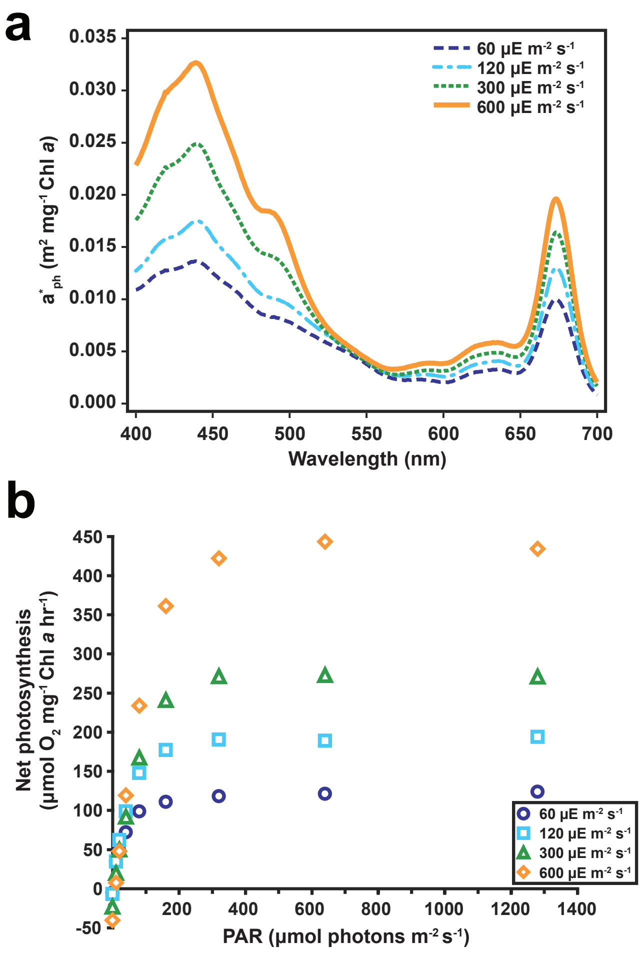

Fig. S7. Relative spectral irradiance of cool white fluorescence light for cultivation and LED light for photosynthesis vs. quantum flux determination.

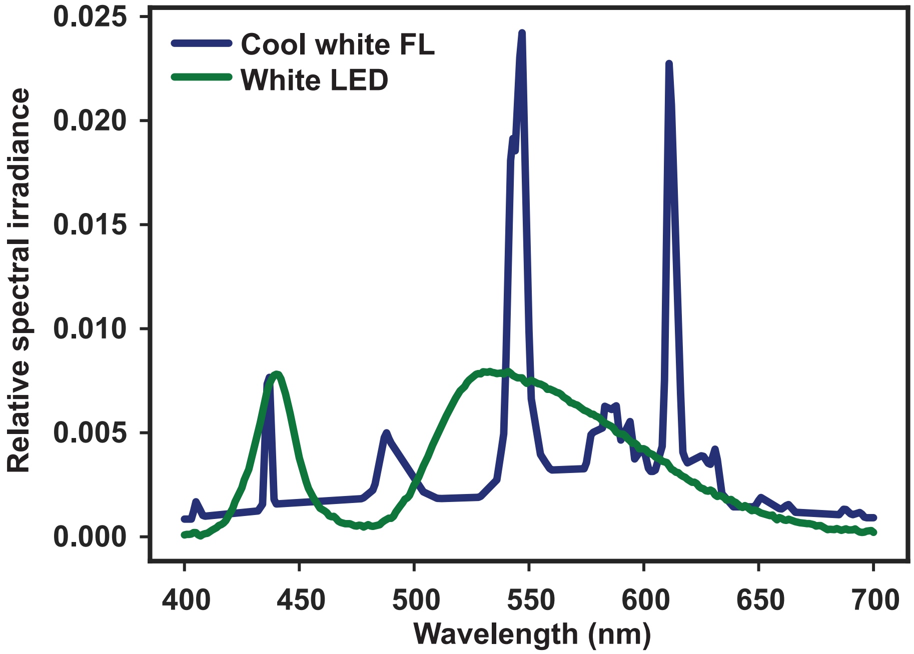

Fig. S8. Co-expression connectivity between GOX_m and KOG annotated pathway clusters.0: Translation, ribosomal structure and biogenesis; 1: Transcription; 2: Posttranslational modification, protein turnover, chaperones; 3: uroporphyrinogen-III synthase (not shown); 4: Intracellular trafficking, secretion, and vesicular transport; 5: Energy production and conversion; 6: Chromatin structure and dynamics; 7: Carbohydrate transport and metabolism; 8: Cell motility (not shown); 9: Cytoskeleton; 10: RNA processing and modification; 11: Amino acid transport and metabolism; 12: Inorganic ion transport and metabolism; 13: Defense mechanisms; 14: Cell cycle control, cell division, chromosome partitioning; 15: Lipid transport and metabolism; 16: Nuclear structure; 17: Cell wall/membrane/envelope biogenesis; 18: Extracellular structures; 19: Nucleotide transport and metabolism; 20: Secondary metabolites biosynthesis, transport and catabolism; 21: Unkonwn (not shown); 22: Signal transduction mechanisms; 23: glutamyl-tRNA reductase (not shown) 24: Coenzyme transport and metabolism; 25: Protoporphyrin IX magnesium chelatase, subunit H (not shown); 26: Photosynthesis; 27: Replication, recombination and repair.

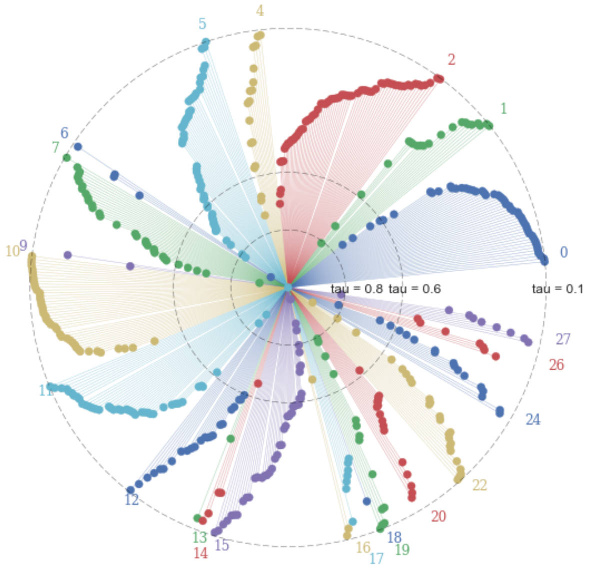

Fig. S9. Energy relocation capacity of the predicted inter-compartment reductant shuttles. (a) TCA cycle. Full oxidation from acetyl-CoA results in 8 electrons transferred to the mitochondrion. (b) Ornithine-glutamine shunt. When operating in the fully cyclic configuration 4 electrons are transferred with no mass loss. Intermediate configurations transfer carbon and nitrogen in addition to electrons. (c) Mitochondrial photorespiratory pathway. The 2 electrons transferred at the glycine cleavage system is divided by two as 2 molecules of glycolate are required to generate the prerequisite 2 glycine molecules. (d) Branched chain amino acid (BCAA) degradation. Acyl-CoA generated by leucine degradation (3 moles acyl-CoA per mole amino acid) and isoleucine degradation (1 mole acyl-CoA per mole amino acid) is not included in the energy relocation capacity. Reaction and metabolite abbreviations are in BiGG format (bigg.ucsd.edu) and correspond to the abbreviations used in the model (Supporting Dataset S1).

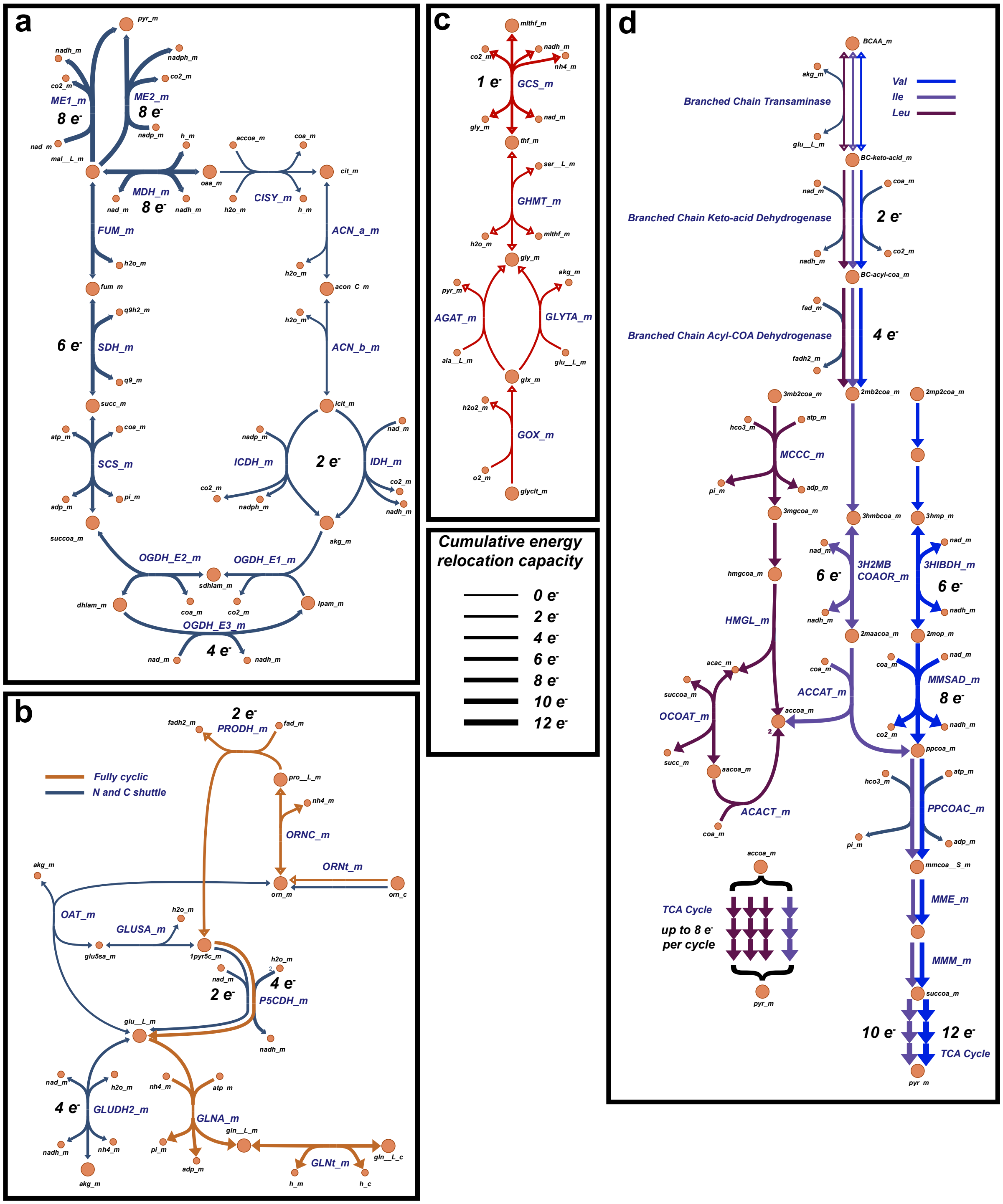

Fig. S10. Gene expression profile of BCAA enzymes from Smith et al. (2016) (20). The low-confidence methymalonyl epimerase (MME, Phatr3_J46728) annotation in the GEM has a similar gene expression profile to high-confidence annotations of BCAA enzymes proponioyl-CoA carboxylase (PPCOAC, Phatr3_J45886 and Phatr3_J51245) and methylmalonyl mutase (MMM, Phatr3_J51830).

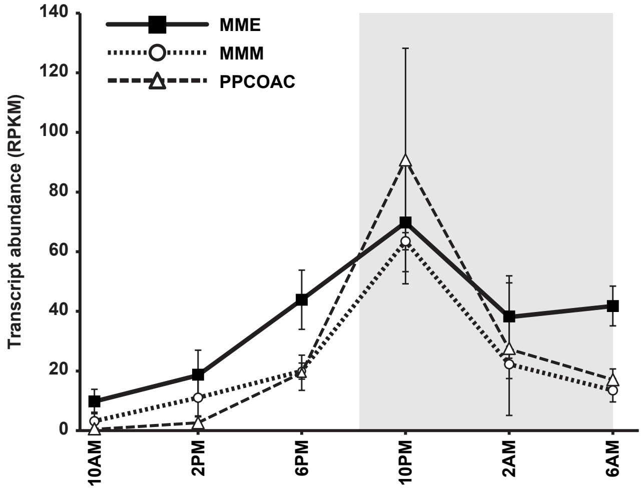

Fig. S11. Hypothetical 2-oxoglutarate shuttle in *P. tricornutum*. (a) Default model simulations for *P. tricornutum* acclimated to low light (60 μmol photons m^{-2} s^{-1}) without a 2-oxoglutarate transporter. (b) Model simulations for *P. tricornutum* acclimated to low light (60 μmol photons m^{-2} s^{-1}) with a 2-oxoglutarate transporter. Fluxes are normalized to 100 units of RubisCO carboxylase flux.

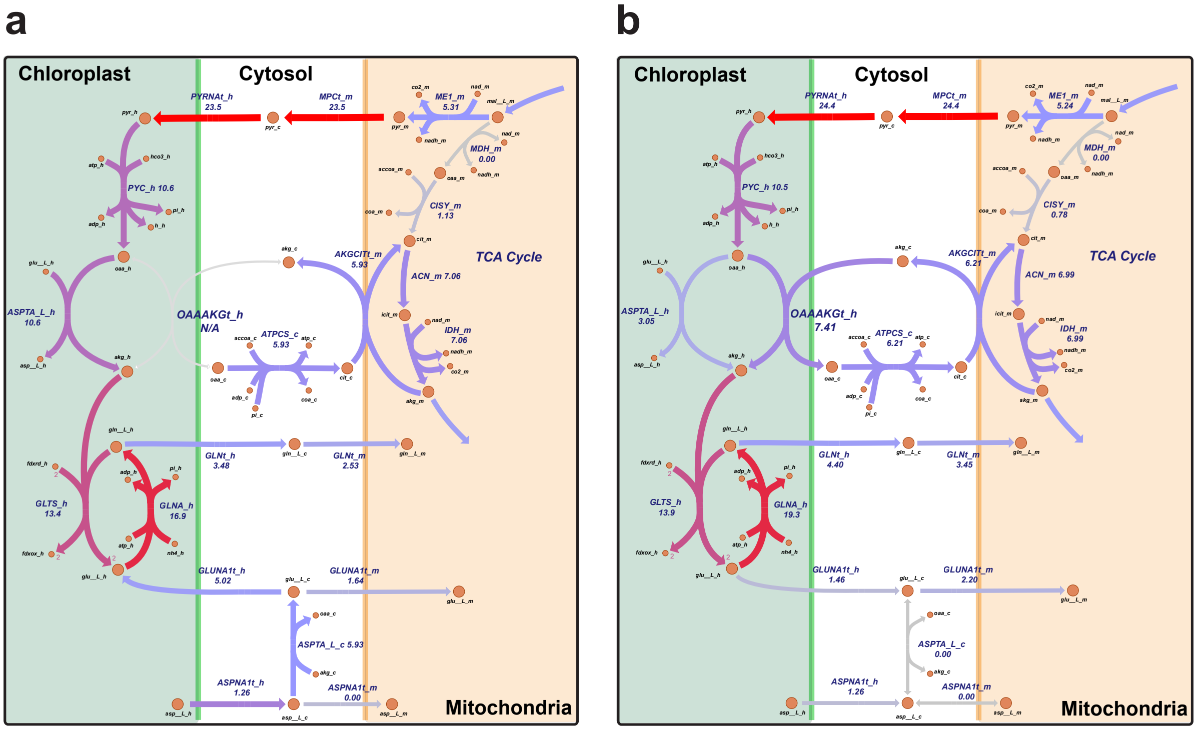

Supplement: Supplementary file 1 — Dataset S1 Modeling files can be found at http://systemsbiology.ucsd.edu/Downloads/SupplementalData. Fig. S1 Biophysical constraints for simulation of P. tricornutum under a sinusoidal light regime. Fig. S2 Comparison of chloroplast volume determined from z‐stacked and 2D confocal microscopy images. Fig. S3 Sensitivity analysis of biophysical constraints for simulations of P. tricornutum under a sinusoidal light regime. Fig. S4 Sensitivity analysis of biomass components for simulations of P. tricornutum under a sinusoidal light regime. Fig. S5 Physiology metrics and simulation comparisons for P. tricornutum acclimated to four PAR values. Fig. S6 Photophysiology metrics for P. tricornutum acclimated to different light intensities. Fig. S7 Relative spectral irradiance of cool white fluorescence light for cultivation and LED light for P vs E measurements. Fig. S8 Coexpression connectivity between GOX_m and KOG annotated pathway clusters. Fig. S9 Energy relocation capacity of the predicted intercompartment reductant shuttles. Fig. S10 Gene expression profile of BCAA enzymes. Fig. S11 Hypothetical 2‐oxoglutarate shuttle in P. tricornutum. Methods S1 Cell cultivation, growth monitoring and physiology. Methods S2 Plastid volume determination. Methods S3 Photophysiology constraints. Methods S4 Photoautotrophic simulations of cellular growth. Methods S5 Membrane inlet mass spectrometry Table S1 Summary of changes in the genome‐scale model of P. tricornutum. Table S2 Dynamic biomass acclimation over the light period in P. tricornutum under different light regimes. Table S3 Chloroplast volume derived from confocal microscopy images of P. tricornutum acclimated to different light conditions. Table S4 Reconstructed carbon balance of the derived model biomass objective function. Table S5 Pigment profiles of P. tricornutum at different acclimated square‐wave light conditions. Table S6 Chloroplast resource allocation as a function of photoacclimated PAR. Table S7 Averages of membra [file NPH-222-1364-s001.pdf]
